# Deep Learning for Hyperpolarized NMR of Intrinsically Disordered Proteins without Resolution Loss: Access to Short‐Lived Intermediates

**DOI:** 10.1002/chem.202502067

**Published:** 2025-08-08

**Authors:** Ertan Turhan, Christopher Pötzl, Dörte Brandis, Federico Faglia, Milan Zachrdla, Dennis Kurzbach

**Affiliations:** ^1^ Faculty of Chemistry, Institute of Biological Chemistry University of Vienna Währinger Str. 38 Vienna 1090 Austria; ^2^ Doctoral School of Chemistry (DoSChem), Faculty of Chemistry University of Vienna Währinger Str. 38 Vienna 1090 Austria

**Keywords:** biomineralization, dissolution DNP, hyperpolarized NMR, machine learning, resolution enhancement

## Abstract

The inherently low sensitivity of solution‐state Nuclear Magnetic Resonance (NMR) has long limited its ability to characterize transient biomolecular states at atomic resolution. While dissolution dynamic nuclear polarization (dDNP) offers a compensating signal enhancement, its broader use has been hindered by rapid polarization decay, causing severe spectral distortion. Here, we introduce HyperW‐Decon, an approach that enables high‐sensitivity, high‐resolution NMR of biomolecules in solution. HyperW‐Decon combines two key aspects: (i) the use of hyperpolarized water (HyperW) to transfer polarization to proteins through rapid proton exchange; and (ii) a theory‐driven, machine learning (ML)–based deconvolution method that corrects polarization‐induced artifacts without requiring external reference signals. This approach is based on a first‐principles understanding of dDNP line shapes and delivers a scalable solution to spectral distortion. Applied to intrinsically disordered proteins (IDPs) involved in biomineralization, HyperW‐Decon reveals previously inaccessible, short‐lived ion‐peptide encounter complexes with residue resolution.

## Graphical abstract

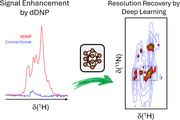

## 1 Introduction

Nuclear Magnetic Resonance (NMR) spectroscopy is a key technique across diverse scientific disciplines, including chemistry, biology, and physics. Its unparalleled ability to resolve molecular structures and dynamics at atomic resolution makes it indispensable for studying complex systems.^[^ ^1^, ^2^, ^3^, ^4^ ^]^ However, NMR is inherently limited by low sensitivity, which significantly hinders its application to low‐concentration molecules or moieties, leaving many biologically and chemically important targets inaccessible.

Dissolution dynamics nuclear polarization (dDNP) promises to overcome this sensitivity barrier.^[^ ^5^, ^6^, ^7^ ^]^ By spin‐hyperpolarizing nuclei at cryogenic temperatures (∼1 K) in frozen solutions *via* microwave irradiation of codissolved radicals, dDNP can boost NMR signal intensities by over four orders of magnitude for heteronuclei and up to 1,000‐fold for protons. Following this hyperpolarization step, the sample is dissolved with a superheated solvent burst and rapidly transferred to a conventional NMR spectrometer for detection in the liquid state under ambient conditions. While powerful, this method has limitations: the harsh dissolution and transfer conditions (pressure jumps of >10 bar; temperature shocks of >300 K) often denature target molecules with labile folds, complexes based on noncovalent interactions, or coordination polymers. The resulting narrow range of amenable molecules/applications severely restricts its utility in biomolecular NMR applications. Similar scope problems also limit other emerging hyperpolarization technologies such as parahydrogen‐based methods^[^ ^6^, ^8^, ^9^ ^]^ or Overhauser DNP.^[^ ^10^, ^11^ ^]^

To bridge this gap, the Hyperpolarized Water (HyperW) methodology was recently developed.^[^ ^12^, ^13^, ^14^, ^15^, ^16^, ^17^, ^18^, ^19^, ^20^, ^21^, ^22^, ^23^, ^24^, ^25^, ^26^, ^27^, ^28^ ^]^ A water pellet is hyperpolarized and subsequently dissolved and transferred to the NMR spectrometer, where it is mixed with a biomolecular target in solution under ambient, mild conditions. The high proton polarization from the HyperW is transferred to the target molecules through chemical proton exchange or possibly even *via* nuclear Overhauser effects (NOE).^[^ ^26^, ^27^ ^]^ This strategy offers two key advantages: (i) the hyperpolarization pool is independent of the target, which is crucial for biomolecules with short relaxation times that are incompatible with traditional dDNP, and (ii) the biomolecular target avoids exposure to the harsh conditions during the dissolution step, preserving structural and functional integrity. Similar approaches using hyperpolarization reservoirs have also recently been applied to monitor small metabolites with potential in clinical magnetic resonance imaging.^[^ ^29^, ^30^, ^31^ ^]^

HyperW has already enabled innovative high‐field applications, such as elucidating membrane interactions,^[^ ^18^ ^]^ studying RNA‐based enzyme kinetics,^[^ ^23^ ^]^ and probing the structural dynamics of unfolding intermediates.^[^ ^24^ ^]^ However, 2D (2D) correlation spectra, the basis of biomolecular NMR, often suffer from resolution penalties in HyperW applications (and dDNP in general).^[^ ^14^, ^17^, ^18^, ^32^ ^]^

**Figure 1:**
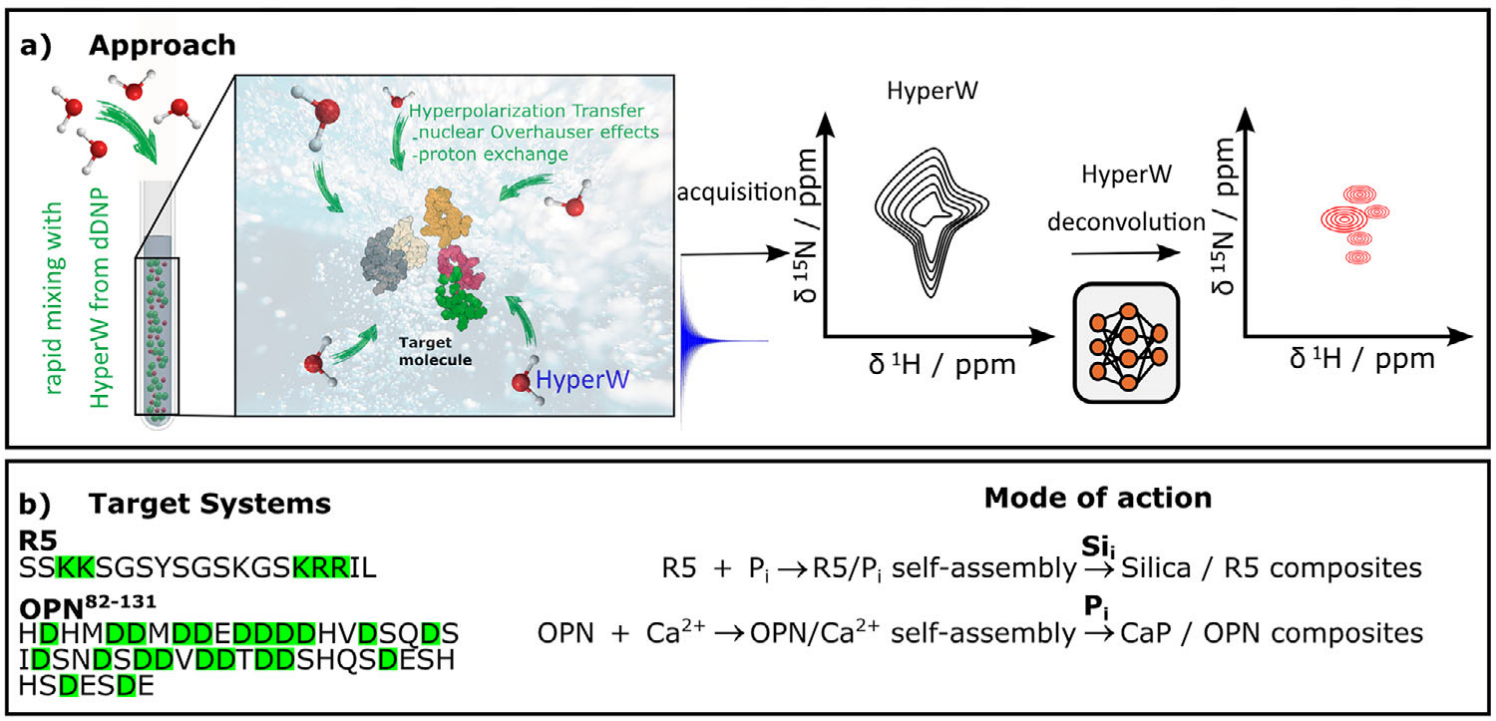
Sketch of the methodological approach and of the studied systems. a) First, hyperpolarized water (HyperW) from a dissolution dynamic nuclear polarization (dDNP) setup is mixed with a protein solution. Proton exchange and NOE transfer the hyperpolarization from HyperW to the target molecule. Then, within the hyperpolarization lifetime of ca. 1 min, a signal‐boosted 2D NMR spectrum of the protein is recorded. Finally, the dDNP‐typical spectral distortions are corrected by a deep‐learning‐based deconvolution approach, resulting in high‐sensitivity and resolved spectra. b) The R5 peptide binds with lysine and arginine side chains (marked green) to inorganic phosphate (P_i_) counterions, triggering self‐assembly into defined structures. These self‐assemblies can scavenge silicate (Si_i_) from the solution and condense it into defined silica nanoparticles. OPN^82‐131^ can bind to Ca^2+^ counterions due to abundant aspartate residues (marked green). The resulting self‐assemblies scavenge P_i_ from solution, leading to biomimetic calcium phosphate (CaP). For both peptides, the structural dynamics behind their biomineralizing activity remain largely undisclosed.

Here, we present a solution to this problem, employing machine learning (ML) algorithms to accurately identify and deconvolute the exogenous HyperW‐induced signal distortions from the true spectral components in hyperpolarized biomolecular 2D NMR spectra. ML has recently found enormous interest in the treatment of NMR data, not least as training sets can be very robustly produced as the physics behind line shapes are well known.^[^ ^33^, ^34^, ^35^, ^36^, ^37^, ^38^, ^39^, ^40^, ^41^, ^42^, ^43^ ^]^ In particular, intriguing approaches have been reported in structure prediction^[^ ^40^, ^44^ ^]^ and line shape improvements^[^ ^45^, ^46^, ^47^ ^]^. Our approach, sketched in Figure 1a, enables the acquisition of orders of magnitude sensitivity‐enhanced 2D correlation spectra of proteins and nucleic acids while not compromising resolution–a methodology that improves signal strength while retaining key advantages of biomolecular NMR that were formerly incompatible with hyperpolarization approaches. Notably, other signal correction methods in, for example, HYPNOESIS, have also been found successful, albeit only with 1D data.^[^ ^31^ ^]^

Other powerful methods, such as ultrafast (UF) NMR in combination with dDNP, might similarly overcome distortions by reducing measurement times to milliseconds. Successful applications have been, for example, demonstrated in diffusion spectroscopy or the detection of metabolites.^[^ ^8^, ^48^, ^49^, ^50^ ^]^ However, despite their potential, such methods have not yet been applied to hyperpolarized high‐resolution protein NMR. On the one hand, they are incompatible with HyperW as the large hyperpolarization reservoir would render gradient encoding challenging. On the other hand, a protein sample can typically not be directly hyperpolarized due to rapid signal loss during sample transfer. The method proposed herein provides a solution to both impasses.

Contrasting such approaches, we develop herein a reference‐free HyperW‐deconvolution (HyperW‐Decon) approach. Unlike conventional reference deconvolution^[^ ^51^, ^52^ ^]^, our method is based on a theoretical model of signal decay in 2D dDNP experiments, explicitly accounting for the convolution of intrinsic relaxation and the hyperpolarization loss throughout the data acquisition process. Based on this distinction, we could derive ML tools to identify the hyperpolarization loss component in the relaxation process and correct the spectra, but without requiring any external reference. This strategy eliminates the need for carefully matched reference signals or simulations, thereby fostering accessibility and applicability across diverse dDNP experiments, including those targeting dynamic and heterogeneous biomolecular systems. As will be shown below, our approach enables high‐resolution NMR of transient biomolecular states previously inaccessible.

We demonstrate the utility of HyperW‐Decon using two intrinsically disordered proteins (IDPs), osteopontin (OPN)^[^ ^53^, ^54^ ^]^ and R5^[^ ^55^, ^56^, ^57^ ^]^, both key players in biomineral formation (Figure 1b). These molecules are scientifically intriguing due to their potential to mediate the shape and morphology of eco‐friendly biominerals. OPN mediates the assembly of calcium phosphate biominerals, while R5 drives the formation of silicate particles, both producing functional, biocompatible structures with potential applications in drug delivery and enzyme encapsulation.^[^ ^58^, ^59^ ^]^ Importantly for their functionality, both peptides self‐assemble into large structures that guide the condensation of oxo‐acid salts—phosphate for OPN and silicate for R5—into well‐defined morphologies.^[^ ^56^, ^57^, ^60^, ^61^, ^62^, ^63^ ^]^ In other words, they have templating functions. In OPN, this process involves the formation of Ca^2+^‐mediated salt bridges between aspartate residues, while in R5, phosphate (P_i_) bridges link lysine residues. The resulting coacervates recruit salts from the surrounding solution, leading to biomimetic nanoparticles. Figure 1b shows a sketch of the biomineralization processes.

With HyperW‐Decon, we access formerly untraceable, short‐lived early‐stage intermediates in the biomineralization processes involving OPN and R5 within seconds after formation.

## 2 Results and Discussion

Our HyperW experiments were conducted using the dDNP prototype described in our earlier work.^[^ ^25^, ^27^, ^32^ ^]^ Briefly, a water pellet containing 10% v/v glycerol‐d_8_ and 15 mM TEMPOL as the polarization agent was hyperpolarized at *T*_DNP_ = 1.4 K in a magnetic field of *B*_0,DNP_ = 6.7 T (more details in the Supporting Information). The hyperpolarized pellet was then dissolved in a 40‐fold excess of D_2_O (an empirically optimized dilution factor^[^ ^27^ ^]^) and transferred to a mixing and injection system adapted from the design described in detail in reference^[^ ^64^ ^]^. This custom injector mixed the HyperW solution homogeneously in a 3:1 ratio with a solution of one of the target IDPs at an initial concentration of 2 mg/mL (before dilution). At the same time, it degassed the mixed solutions to reduce sample heterogeneity. Additional experimental details are available in the Supporting Information.

Following the mixing process, the dDNP prototype promptly initiated the acquisition of BEST‐HMQC (band‐selective excitation, short transient‐heteronuclear multi‐quantum coherence) spectra^[^ ^65^ ^]^, using the pulse sequence described by Kaderávek et al. (all details in reference.^[^ ^16^ ^]^) The spectra were recorded with 64 complex *t*_1_ increments, each consisting of two scans, separated by a recycling delay (τ) of 100 ms plus 100 ms for the acquisition of the free induction decay (FID). The total acquisition time of each spectrum was ca. 50 s.

While signal enhancements of several orders of magnitude were achieved for both IDPs (*vide infra*), the key problem remained. Throughout the acquisition period, the HyperW polarization decayed, resulting in a nonconstant signal boost during the encoding of the indirect dimension and a prohibitively strong line broadening in the indirect ^15 ^N dimension.

**Figure 2:**
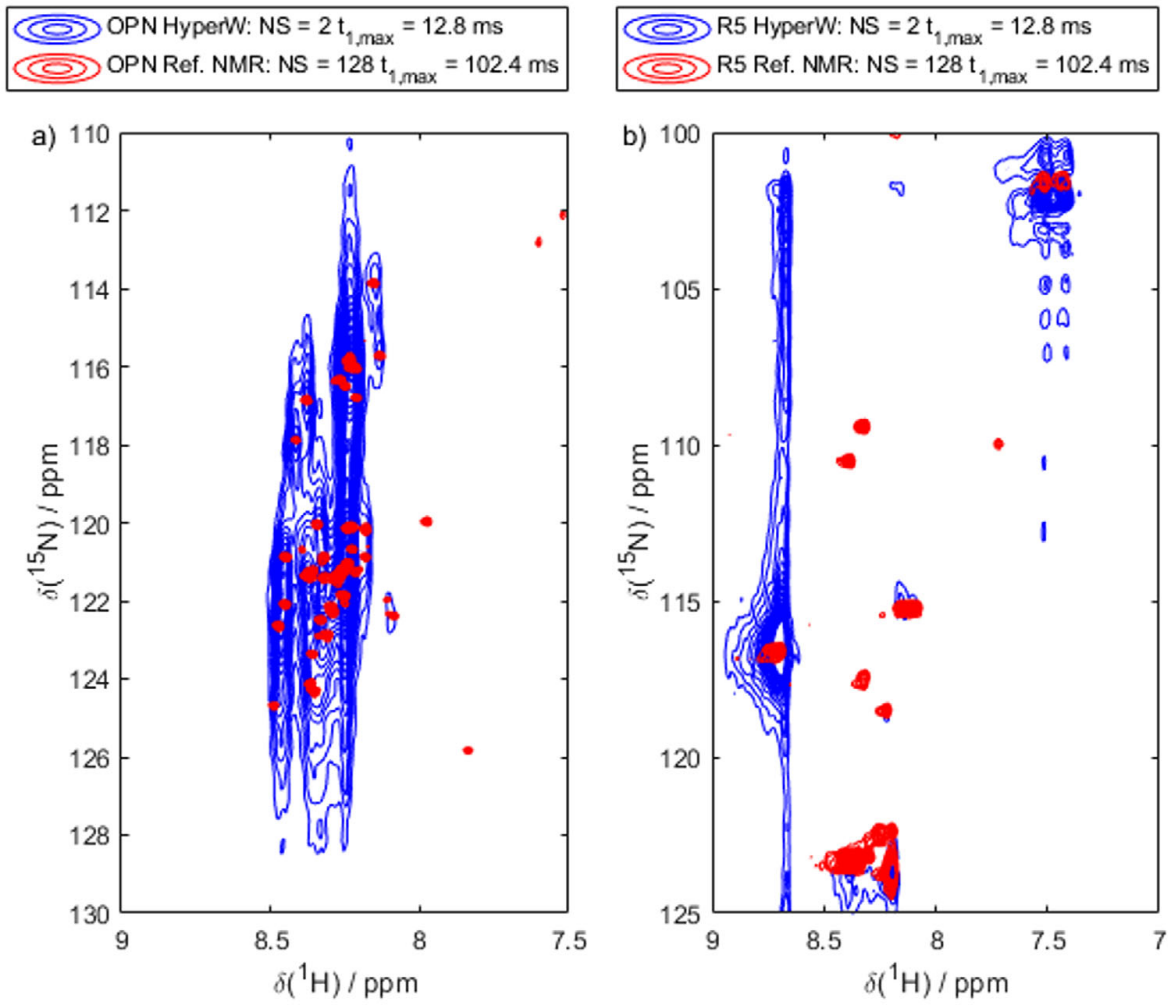
The key problem: Broadening induced by HyperW. a) ^1^H‐^15^N HMQC of OPN^82‐131^ (Ca^2+^ complex) in HyperW (blue; 2% hyperpolarized H_2_O) and after decay of the hyperpolarization. Evidently, the hyperpolarized spectrum is significantly broadened in the ^15^N dimension, notwithstanding much stronger signal intensities. b) ^1^H‐^15^N HMQC of R5 in HyperW (blue) and a conventional buffer (red). Again, strong broadening by HyperW is evidenced. Note that the apparent line broadening along the ^1^H‐dimension for the very intense peak was also observed in the reference recorded in thermal equilibrium and is not due to temperature instabilities.

Figure 2 demonstrates this problem. It shows HyperW spectra of the calcium‐binding domain of OPN (amino acids 82–131; henceforth denoted OPN^82‐131^) and R5 in comparison to their conventional counterparts (note that in all figure legends, NS denotes the number of averaged scans per FID, and *t*_1,max_ denotes the maximum evolution time of the FID along *t*_1_, recorded with a time increment of 0.2 ms; all spectra can be found in enlarged representations in the Supporting Information as well). The signal intensity is much better with HyperW, but the hyperpolarization decay causes excessive spectral distortions. This exogenous signal broadening and its cancellation lie at the center of our proposed hyperpolarized water deconvolution method, which aims to recover native, non‐biased line shapes while retaining the signal enhancement. Note that each figure caption explicitly states the water content of the used buffers.

### 2.1 Data Treatment: Rationale and Theory

We considered that during the encoding of the indirect dimension in the BEST HMQC (*i.e*., along the *t*_1_ dimension), every increment starts with a different starting polarization as opposed to conventional NMR in thermal equilibrium. The polarization of the detected backbone amide ^1^H^N^ nuclei is replenished between each increment^[^ ^66^ ^]^ mainly by chemical exchange with hyperpolarized solvent protons instead of nuclear relaxation. With increasing *t*_1_, the number of replenished nuclei decreases due to the decay of the HyperW polarization toward thermal equilibrium polarization.

As a result, the intrinsic FID along the indirect dimension (denoted *I*(*t*_1_)) is multiplied with an exogenous exponential decay function *E*(t_1_). The recorded signal for each resonance along the ^15 ^N dimension under HyperW conditions *S*(*t*_1_) can then be described as:

(1) $$S\left(t_{1}\right)=E\left(t_{1}\right)\cdot I\left(t_{1}\right)$$

with

(2) $$E\left(t_{1}\right)=\varepsilon \cdot \exp\left(-R_{1,\mathrm{ef}\;\;f}\cdot t_{1}\right)$$

where ε is the signal enhancement of the protein signal at *t*_1_ = 0, and *R*_1,eff_ the effective additional relaxation rate constant of the amide resonances induced by the exchange with HyperW. *R*_1,eff_ is related to the intrinsic longitudinal relaxation of HyperW but is not equal. It is shorter as HyperW experiments are not recorded with any dummy scans, causing an effectively accelerated loss of the starting polarization.^[^ ^27^ ^]^ Note that the effective FIDs do not decay with a single cumulative decay rate, but biexponentially, with the water decay function being multiplied with the intrinsic FID. While the latter is changing from peak to peak, the former remains constant due to the intrinsically disordered nature of the probed proteins; thus, it can be identified and approximated with a single value. Note further that for *E*(t_1_) the thermal polarization has been neglected due to the large water hyperpolarization. Thus, this function has no offset. For small signal enhancements, an offset might yet be necessary.

**Figure 3:**
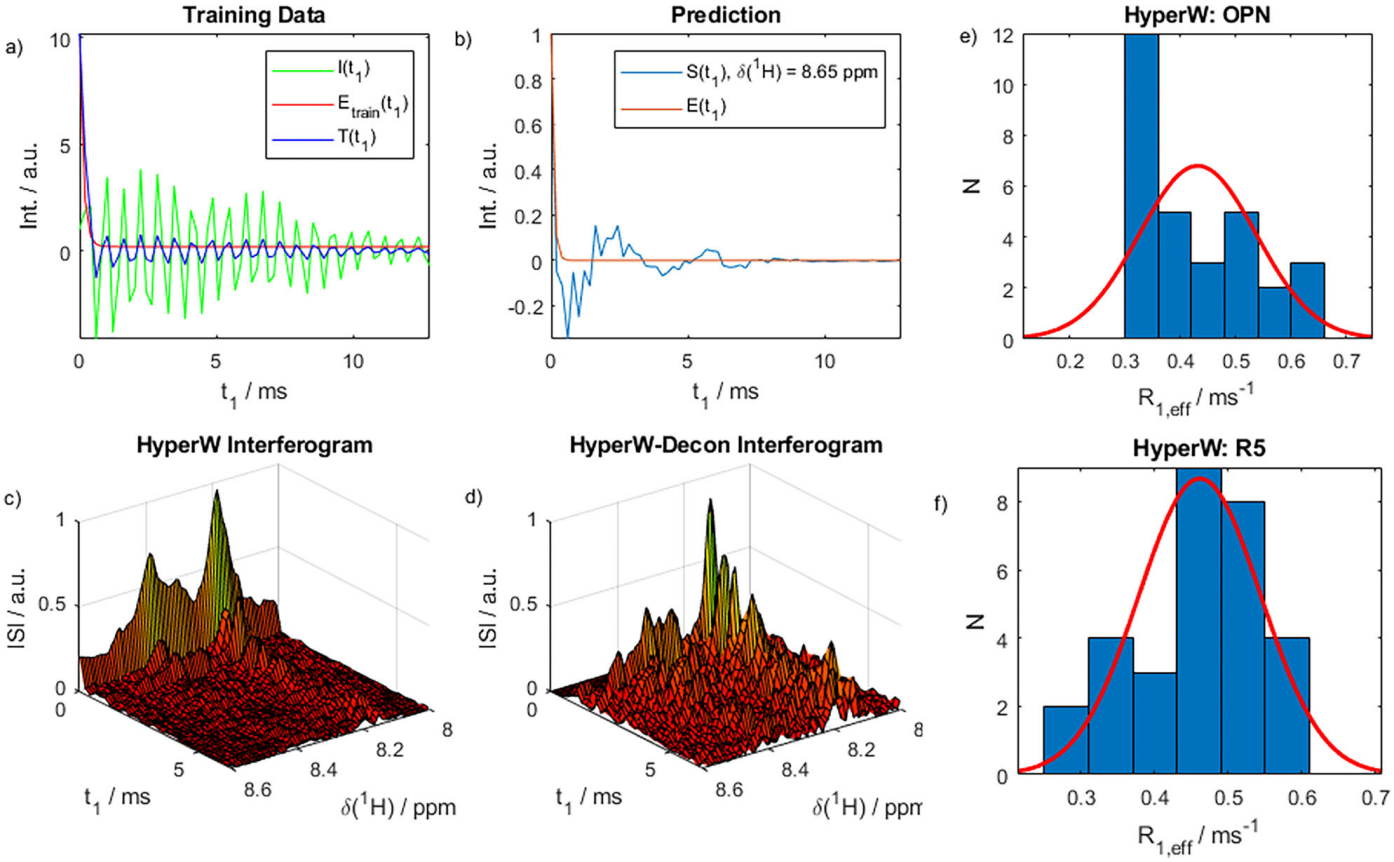
HyperW deconvolution. a) Exemplary training data for the neural networks. An actual experimental FID along *t*_1_ (real part) in thermal equilibrium (green), a simulated HyperW decay function (red), and the multiplication of both (blue) used for training are shown. In total, 100,000 such functions were used for training. b) Experimental FID from a HyperW experiment on R5 along *t*_1_ (blue) and the exponential function identified by the neural network (orange). c,d) Exemplary interferograms of R5 before and after deconvolution (data truncated at *t*_1_ = 6 ms for clarity). e, f) Rates of exogenous decay functions *E*(*t*_1_) predicted by the neural networks for OPN^82‐131^ and R5 for 50 FIDs. The mean of the distributions was used for further data treatment downstream.

If the exogenous decay function *E*(t_1_) is identified, the recorded FIDs can be corrected, that is, divided by the spurious decay. This operation corresponds to a deconvolution of the spectra after Fourier transform (FT) along the ^15 ^N dimension. Figure 3a‐d visualise this process.

*E*(t_1_) is, yet, complex to determine as the exchange with the target molecule, the abovementioned lack of dummy scans, as well as the overlap between the water signal and the excitation pulse profile, influence the hyperpolarization lifetime.^[^ ^27^ ^]^ However, one can make use of the fact that the same exogenous decay function modulates all FIDs along *t*_1_, as the water hyperpolarization that mediates the signal enhancement decays with a single rate constant to thermal equilibrium.^[^ ^13^, ^66^ ^]^ Therefore, the decay function *E*(t_1_) can be considered a recognizable common feature of every FID. To identify the relevant decay function, we trained, validated, and tested DNNs with 10^5^ simulated FIDs derived from our thermal equilibrium data *I*(*t*_1_). The training FIDs denoted (*T*(*t*_1_)) were of the form (cf. Figure S1):

(2) $$T\left(t_{1}\right)=I\left(t_{1}\right)\cdot \varepsilon_{\mathrm{train}}\exp\left(-R_{1,\mathrm{train}}\cdot t_{1}\right)+\mathrm{noise}$$

where the training enhancements and relaxation rates ε_train_ and *R*_1,train_ as well as noise levels were varied. The resulting trained networks were then given the hyperpolarized FIDs *S*(*t*_1_) to predict *R*_1,eff_. (Note that due to the high levels of polarization, the water signal can be modeled to decay to naught, as the thermal equilibrium value becomes negligible and fades into the baseline of the intrinsic FID.) The DNN did not engage in any further signal refinement. It only determined the rate constant *R*_1,eff_. Further details and methodological considerations can be found in the Supporting Information.

Interestingly, due to the straightforward nature of the problem, training with 10^5^ FIDs already sufficed for correct predictions. To remove the exogenous HyperW component from the FIDs, we then divided *S*(*t*_1_) by the predicted function *T*(*t*_1_)/*I*(*t*_1_) before Fourier Transform along *t*_1_. This approach was inspired by the reference deconvolution approach originally developed by Morris, Webb, and others.^[^ ^51^, ^52^ ^]^

Reference deconvolution is a signal processing technique in NMR spectroscopy that enables the correction of line shape distortions arising from instrumental imperfections or intrinsic interactions, thereby recovering high‐resolution spectral features. The method models the experimental signal as a convolution of the ideal spectrum with a known response function, often derived from a reference signal.^[^ ^51^, ^52^ ^]^ By deconvolving this known response, one can sharpen the lines, improve peak position accuracy, and suppress spectral artifacts. This technique has been applied extensively in multidimensional NMR to counteract inhomogeneous broadening and to reconstruct well‐defined resonance structures in crowded spectra.

Recent developments have significantly expanded the utility of reference deconvolution, especially when integrated into nonuniform sampling (NUS) and compressed sensing (CS) workflows. Kazimierczuk et al.^[^ ^67^, ^68^ ^]^ embedded virtual decoupling directly into the CS reconstruction of 3D HNCA spectra, enabling resolution of overlapping resonances in large, disordered proteins like Tau and photoreceptors without additional experimental time. Such integration reduces acquisition demands while enhancing both resolution and sensitivity. Other examples include region‐selective deconvolution, where the correction is confined to specific spectral zones—for example, suppressing glycine‐induced broadening—thus minimizing reconstruction artifacts and improving signal fidelity. In parallel, ML approaches like RH‐Unet have emerged^[^ ^69^ ^]^, training neural networks to reverse distortions introduced by inhomogeneous magnetic fields based on reference peak behavior, outperforming traditional deconvolution in complex systems.^[^ ^70^ ^]^

Our deconvolution workflow, together with the results of the two trained DNNs for both peptides, is shown in Figure 3e–f. Based on our theoretical understanding of the HyperW experiment, we could develop a DNN approach that is fully automated and parameter‐free from the user's perspective, reducing both subjectivity and operator time. Traditional reference deconvolution methods typically require manual tuning, residual inspection, or multi‐start optimization, which we found impractical for our various dDNP applications.

In the following, we will first discuss the results of this approach for OPN^82‐131^ and R5 and then demonstrate its use in biomineralization applications of both peptides.

### 2.2 Osteopontin

**Figure 4:**
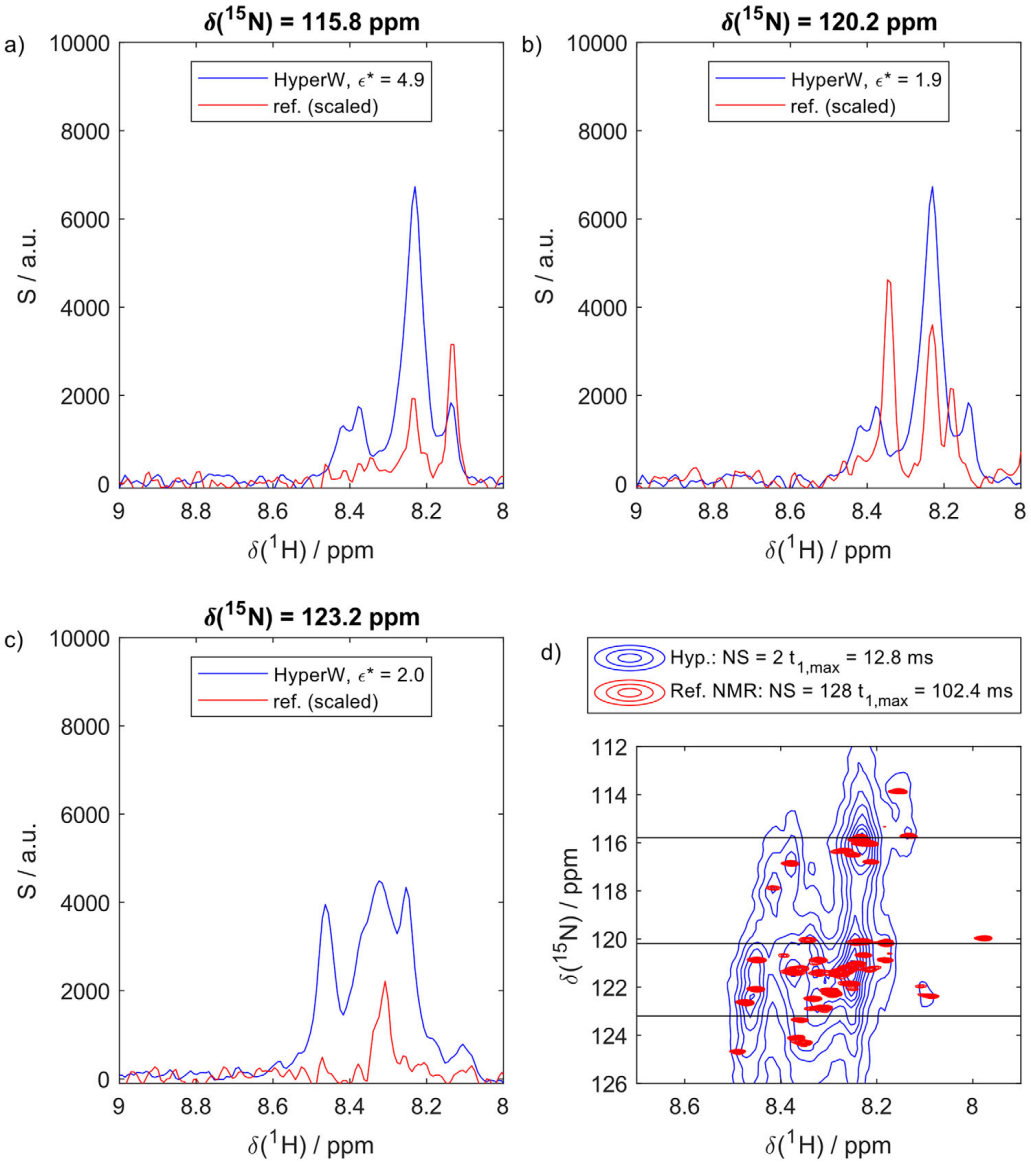
Signal‐enhancements in HyperW‐NMR of OPN^82‐131^. a‐c) Slices through the recorded 2D HMQC spectrum in HyperW (blue; 2% H_2_O) and a reference spectrum in conventional water (red; 2% H_2_O), scaled to the same noise level. The signal enhancement is observable. d) A 2D ^1^H‐^15^N HMQC in HyperW and in conventional water. The slices shown in panel (a) are indicated as black lines.

We started our investigation with OPN, which was also subject to our earlier work. Hence, its behavior under HyperW conditions is well documented.^[^ ^14^, ^20^, ^71^, ^72^, ^73^ ^]^ First, we evaluated the signal enhancements in the 2D spectra before engaging in any peak shape optimization. Figure 4a–c shows slices through the 2D ^1^H‐^15 ^N BEST‐HMQC spectrum of OPN^82‐131^ in a HyperW‐based buffer (blue) and compares them to spectra recorded in conventional solution (red). We chose the Ca^2+^ complex of OPN as a starter model to develop our method, as it led to very strong signal intensities. The hyperpolarized spectrum was recorded within 50 s, while the conventional (meaning all polarizations in thermal equilibrium) counterpart was recorded in 80 min. Despite this difference, the signal‐to‐noise ratios (SNR) in these slices were between 10 and 50 in the hyperpolarized case but consistently below ∼10 in the reference. Considering that the SNR depends on the square root of the measurement time, this corresponds to signal enhancements ε between 130 and 650, hence, an improvement by over two orders of magnitude and close to what can be achieved with proton‐based dDNP. Figure 4c indicates where these slices are located in the spectra (blue: recorded in HyperW, red: recorded in thermal equilibrium). The SNR enhancement translates into “improvement” factors ε* of ∼2‐5 (Figure 4a–c), which were defined earlier (all details in ref. [16]) as the plain ratio in SNR between the HyperW experiment and the best spectrum obtainable by conventional NMR (SNR_HyperW_/SNR_Conventional_), not considering concentrations, number of scans, acquisition times etc.

For the presented case, this means that ε* describes not the conventional signal enhancement (normalized to number of scans, receiver gain, etc.), which reflects the high polarization in dDNP experiments, but the mere improvement of the SNR over the best reference spectrum to be obtained under our experiment conditions (see the Supporting Information for more details).

The conventional enhancements ε reported above were evaluated with the exact same sample and detection sequence used for dDNP detection (2% H_2_O / 98% D_2_O due to the HyperW conditions), but after decay of the hyperpolarization. The ‘best’ reference NMR spectrum to compute ε* was obtained with more scans, higher receiver gain, and optimized *t*_1,max_. To evaluate ε* the 2% water sample was consistently taken. The ε* factors are indicated on top of each panel showing 1D slices. ε* translates into the traditional ε value by a factor of 181 (ratio of the number of scans, receiver gain, and considering changing *t*_1,max_, see the Supporting Information). Additionally, some spectra were also recorded in 90% H_2_O to assess the influence of signal attenuation due to deuteration, as expected, a 90/2 = 45‐fold increase in intensity resulted.

Despite this significant sensitivity improvement, it is also evident from the spectra in Figure 4c that the resolution penalty in the HyperW experiment is immense. While the conventional spectrum leads to well‐distinguishable peaks, the HyperW‐based acquisition entailed a prohibitive line broadening along the indirect ^15^N‐dimension. This observation can also be made with other dDNP systems and other approaches to HyperW.^[^ ^14^, ^17^, ^18^, ^22^ ^]^ For OPN^82‐131^, a residue‐resolved analysis, the key approach in biomolecular NMR, was, in fact, entirely impeded by the strong overlap between the different signals.

**Figure 5:**
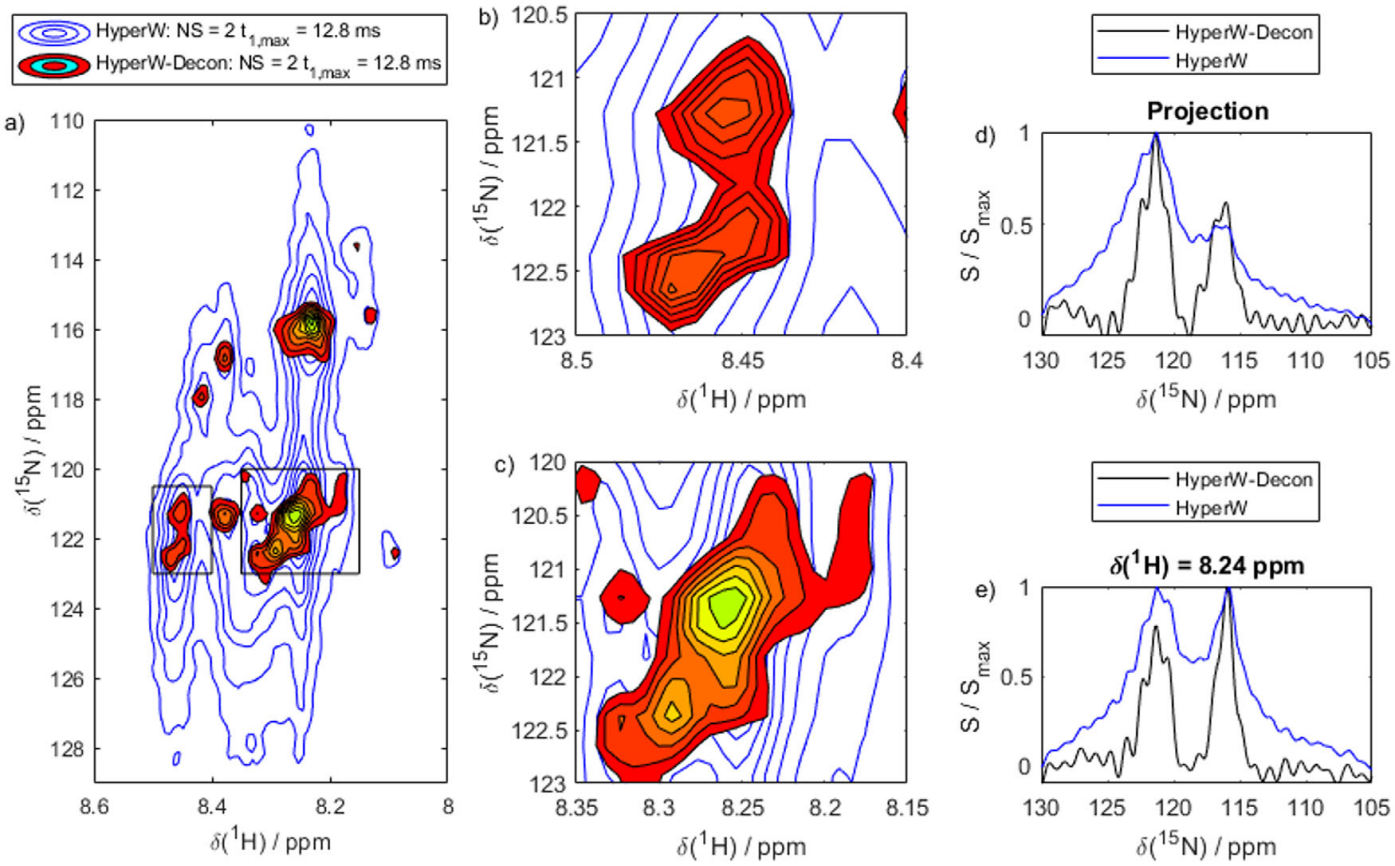
Effect of hyperpolarized water deconvolution. a) A HyperW spectrum (blue; 2% H_2_O) and the corresponding HyperW‐deconvoluted spectrum (red/green). b,c) Zoom on crowded regions of panel (a), indicated by the black rectangles. Individual peaks become distinguishable after deconvolution, where no residue‐resolved analysis was possible before. d) Skyline projection of the Hyper W (blue) and HyperW‐Decon (black) spectrum along the ^15^N dimension showing the effect of the HyperW deconvolution. e) Slice through the Hyper W (blue) and HyperW‐Decon (black) spectrum. Data are normalized to the maximum signal intensity *S_max_*.

To tackle this problem, we then applied the above‐outlined HyperW‐Decon approach to the hyperpolarized spectrum. The results are shown in Figure 5. The deconvolution procedure resulted in the red/green spectrum in Figure 5a,b superposed on the original spectrum (blue). Clearly, the line shape was much improved. While no resolved peaks could be analyzed before, this immediately becomes possible after deconvolution with the decay function *E*(*t*) predicted by our DNN. In Figure 5c, we show a projection and a slice of the 2D spectrum before and after deconvolution. Clearly, the line width is much narrower after the deconvolution procedure.

Our neural network predicted the exogenous decay rates along the indirect dimension to be ∼0.45 ms^−1^ on the *t*_1_ time scale (cf. Figure 3e). Two pieces of information need to be considered when interpreting this value. (*i*) It should not be confused with the actual relaxation rate constant on the experimental time scale (wall time) *t*_exp_ as the FID is recorded as a function of *t*_1_ increments. (*ii*) As explained in the Theory section, it should not be set equal with the intrinsic longitudinal HyperW relaxation, which is on the order of 0.1 s^−1^ (see the Supporting Information Figures. S2–S7 and Figures S8, S9 for additional visualizations of the enhancements). Instead, it reflects the rate with which the FID decays on a time axis ranging from zero to *t*_1,max_.

Further, note that only a subset of residues are detected with every HyperW‐approach^[^ ^27^ ^]^ compared to the full spectrum in Figure 5. It is important to stress that this is not a result of the deconvolution but inherent to HyperW experiments. This is a fundamental feature of the HyperW method. As hyperpolarization is primarily transferred via proton exchange from the solvent to the target protein, the signal enhancement depends strongly on the residue‐specific amide proton exchange frequency (see references^[^ ^16^, ^17^, ^27^ ^]^). Hence, only those residues with especially fast exchange rates, with *k*_ex_>2 s^−1^, become efficiently hyperpolarized. This effect has, for example, been used to map solvent‐exposed surface residues.^[^ ^16^, ^17^ ^]^

**Figure 6:**
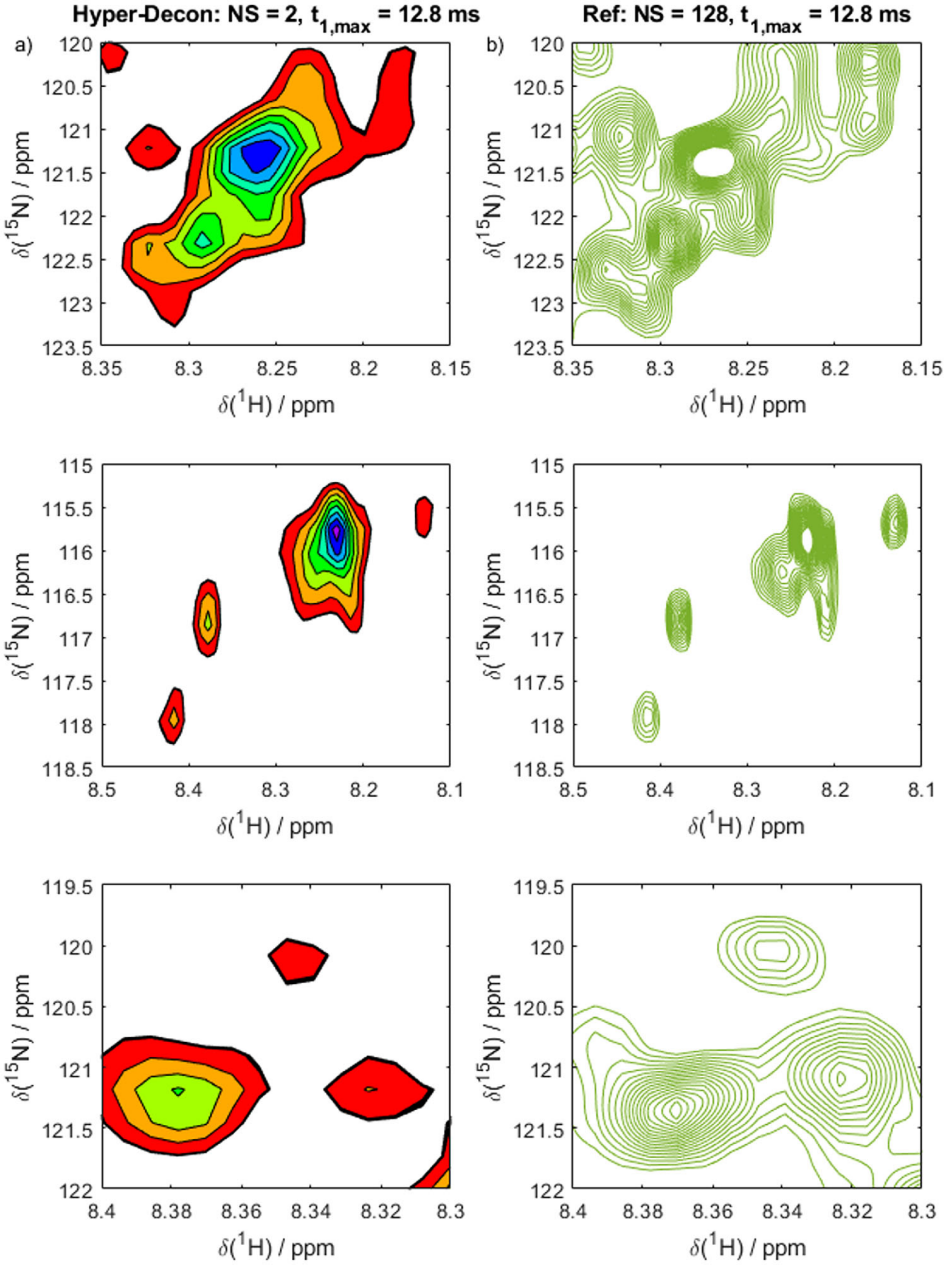
Resolution achieved by the HyperW‐Decon approach. a) Zooms on three different regions of the deconvoluted spectrum (2% H_2_O). b) Zoom on a reference spectrum (90% H_2_O). recorded precisely as the HyperW spectrum, but with 128 scans instead of 2. The match between the peak positions in the hyperpolarized spectrum and the reference confirms that the deconvolution approach correctly produces resonance frequencies.

To validate the HyperW‐Decon spectra, Figure 6a,b compares the deconvoluted spectrum with a conventional HMQC recorded in thermal equilibrium with precisely the same experimental parameters but averaged over longer periods (128 instead of 2 scans). The positions of all deconvoluted peaks are correctly reproduced, as evidenced by their positions relative to the peaks in the reference spectrum. This demonstrates the accuracy of the computed resonance frequencies. Notably, with respect to the resolution, the HyperW‐deconvolution approach is on par with the conventional counterpart.

### 2.3 R5

**Figure 7:**
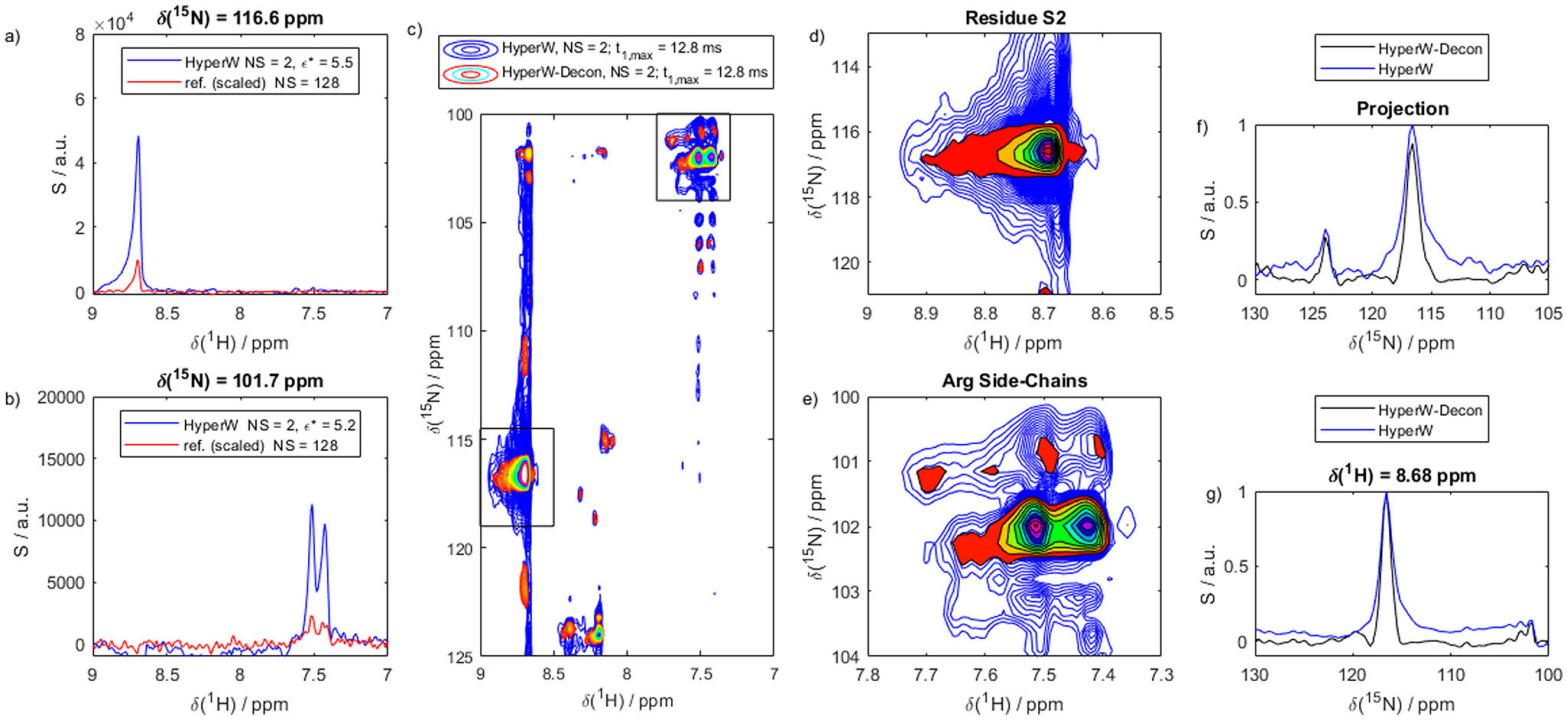
HyperW NMR on R5. a, b) Examples of slices through a HyperW ^1^H‐^13^C HMQC. Blue: hyperpolarized spectrum recorded with 2 scans (2% H_2_O). Red: conventional spectrum recorded with 128 scans (2% H_2_O), scaled to the same noise level c) The 2D HMQC (blue) and the corresponding hyperpolarized water deconvoluted spectrum (red‐green) In particular, for residue S2 and the arginine sidechains (marked with the black rectangles) the effect is significant. d, e) Zoom in the marked regions in panel (b). f) Skyline projection of the spectra along the ^15^N dimension showing the effect of the HyperW deconvolution. g) Slice through the spectra along the ^15^N dimension showing the effect of the HyperW deconvolution.

To further demonstrate the robustness of our method, we tested it with an entirely different IDP, namely R5. The peptide is 19 amino acids long, but it plays a prominent role in directing silica morphogenesis under mild, biotechnologically appealing conditions.^[^ ^55^, ^56^, ^57^, ^58^, ^59^, ^74^, ^75^, ^76^ ^]^ R5 is much shorter than OPN^82‐131^ and has a relatively low primary sequence heterogeneity (see Figure 1). Nonetheless, the experimental approach was the same. The corresponding results are shown in Figure 7. Again, the SNR enhancements scaled between ca. 100 and 500, as evidenced by slices through the hyperpolarized and conventional 2D spectra (Figure 7a,b). The resolution in the HyperW‐boosted spectra was severely compromised (Figure 7c–e), particularly residue S2, as well as the arginine side chains experienced very strong signal enhancements that led to artifacts spanning the entire ^15 ^N spectral width.

Importantly, in this second markedly different example, too, the HyperW‐Decon approach provided satisfying results, sharpening the spectra to adopt native line shapes. The resulting spectra are superposed in the original HyperW results in Figure 7c–e in red/green. Where peaks were broadened such that a detailed analysis was impossible in the HyperW experiment, the deconvolution led to well‐defined signals. As for the previous experiment, the effect is visualized in Figure 7f,g through the 1D projection of the ^15 ^N dimension and the additional slices through the spectrum. Note that the comparison with the thermal equilibrium counterpart also shows a correct identification of the resonance frequencies for all deconvoluted signals (see the Supporting Information).

### 2.4 Application to Biomimetic Material Formation

At this point, we can state that the HyperW‐Decon approach reliably produced high sensitivity and resolved spectra. However, the robustness of the approach and its potential to make novel applications possible still need to be tested. In the following, we demonstrate this in interaction monitoring experiments.

Both OPN^82‐131^ and R5 play critical roles in biomineralization processes and are key to many biomimetic materials design programs. Hence, probing their interactions with mineral salts and the underlying structural dynamics is of high timely interest.^[^ ^53^, ^54^, ^59^, ^61^, ^77^ ^]^

As stated above, the self‐assembly of these peptides is essential for their function as mineral salt scavengers. Yet, despite their importance, the mechanisms underlying their biomineralizing activity remain largely undisclosed. In particular, complex interaction cascades with multiple transient intermediate steps leading to the final mineral particles are not readily accessible by established techniques.^[^ ^60^, ^62^ ^]^

To address this, we applied our newly developed methodology to investigate the short‐lived counterion‐peptide encounter complexes that initiate the first steps of the biomineralization cascades of R5 and OPN.^[^ ^53^, ^54^, ^59^, ^60^, ^61^, ^63^, ^77^ ^]^ Thanks to the strong signal boost and the resulting possibility of recording 2D NMR correlation spectra in less than a minute with high SNR, we conceived an experiment where such short‐lived intermediates become accessible by NMR.

Our experimental approach involved supplementing HyperW with high concentrations of salts that trigger self‐assembly (Ca^2+^ for OPN and P_i​_ for R5) and coinjecting them into peptide solutions waiting in the NMR spectrometer–similar to the strategy successful in earlier studies of biomineralization events by dDNP.^[^ ^78^, ^79^, ^80^, ^81^ ^]^ This strategy allowed for the acquisition of BEST‐HMQC spectra immediately after the encounter of peptides with their respective counterions while dissolved in hyperpolarized buffers.

For both OPN and R5, well‐resolved spectra were obtained following HyperW deconvolution, with residue‐specific chemical shifts clearly identified. These results were unattainable from the raw HyperW data, where severe signal overlap precluded unambiguous interpretation (see the Supporting Information for all data).

**Figure 8:**
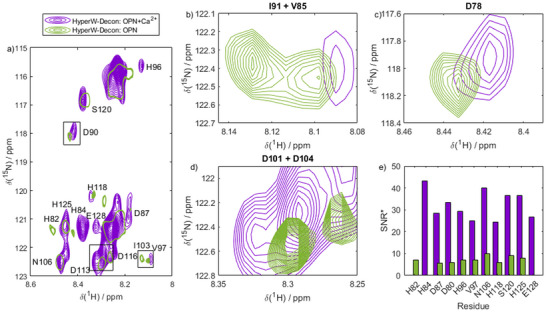
Monitoring OPN‐Ca^2+^ binding by HyperW within 60 s. a) HyperW deconvoluted spectra of OPN in the presence of Ca^2+^ (purple) and in the absence of any multivalent counterions (green, single contour). Spectra were recorded immediately after mixing the IDP with counterions/HyperW. Chemical shift changes and intensity changes can be observed as a result of Ca^2+^ binding. b‐d) Zoom on selected resonances showing that residue‐resolved chemical shifts can be followed with HyperW‐deconvolution, which wasn't possible with conventional dDNP or NMR before. e) Ca^2+^‐dependent signal‐to noise ratios after deconvolution (SNR*). In the presence of Ca^2+^ (purple), the SNR is significantly higher for most residues than in the absence (green) of any bivalent counterions.

For OPN, the interaction with Ca^2+^‐counterions induced heterogeneous spectral changes across the entire spectrum in the HyperW experiments, as shown in Figure 8a. While some resonances remained unaffected, others shifted in frequency or split upon counterion exposure. Notably, peak splitting was predominantly observed in the N‐terminal aspartate‐rich region (residues 86–95), which is known to bind Ca^2+^.^[^ ^61^ ^]^ Figure 8b–d highlights various residues as examples, showing their behavior before and immediately after Ca^2+^ encounter. The hyperpolarized deconvolution approach enabled unambiguous identification and analysis of these residues, even when their signals were closely spaced. Taken together, these changes clearly indicate Ca^2+^ binding.

While chemical shift changes and splitting observed upon Ca^2+^ binding are expected and provide useful insights, the signal‐to‐noise ratios after deconvolution (denoted as SNR*, distinct from the SNR before deconvolution) reveal more intriguing aspects of the presented methodology. Figure 8e highlights SNR* values for strongly enhanced residues distributed throughout the entire primary sequence, showing a nearly homogeneous increase post‐Ca^2+^ exposure, with the exception of residue H82. This observation suggests that Ca^2+^ binding enhances the interaction with the hyperpolarized water, likely through accelerated proton exchange dynamics before engaging in self‐assembly.

This finding aligns with previous reports showing a tendency of OPN to form compact states stabilized by intramolecular electrostatic interactions, which reduce solvent exposure.^[^ ^71^, ^72^ ^]^ Upon exposure to high ionic strengths or denaturing agents such as urea, these compact cores dissolve, leading to an expansion of the IDP into a more elongated, solvent‐accessible conformation.^[^ ^71^, ^72^ ^]^

Our results suggest a similar interpretation: Ca^2+^ binding alters intramolecular electrostatic potentials, disrupting the compact core and increasing solvent exposure of the peptide. This enhanced solvent interaction fosters hyperpolarization transfer, as reflected in the observed increase in SNR*.

**Figure 9:**
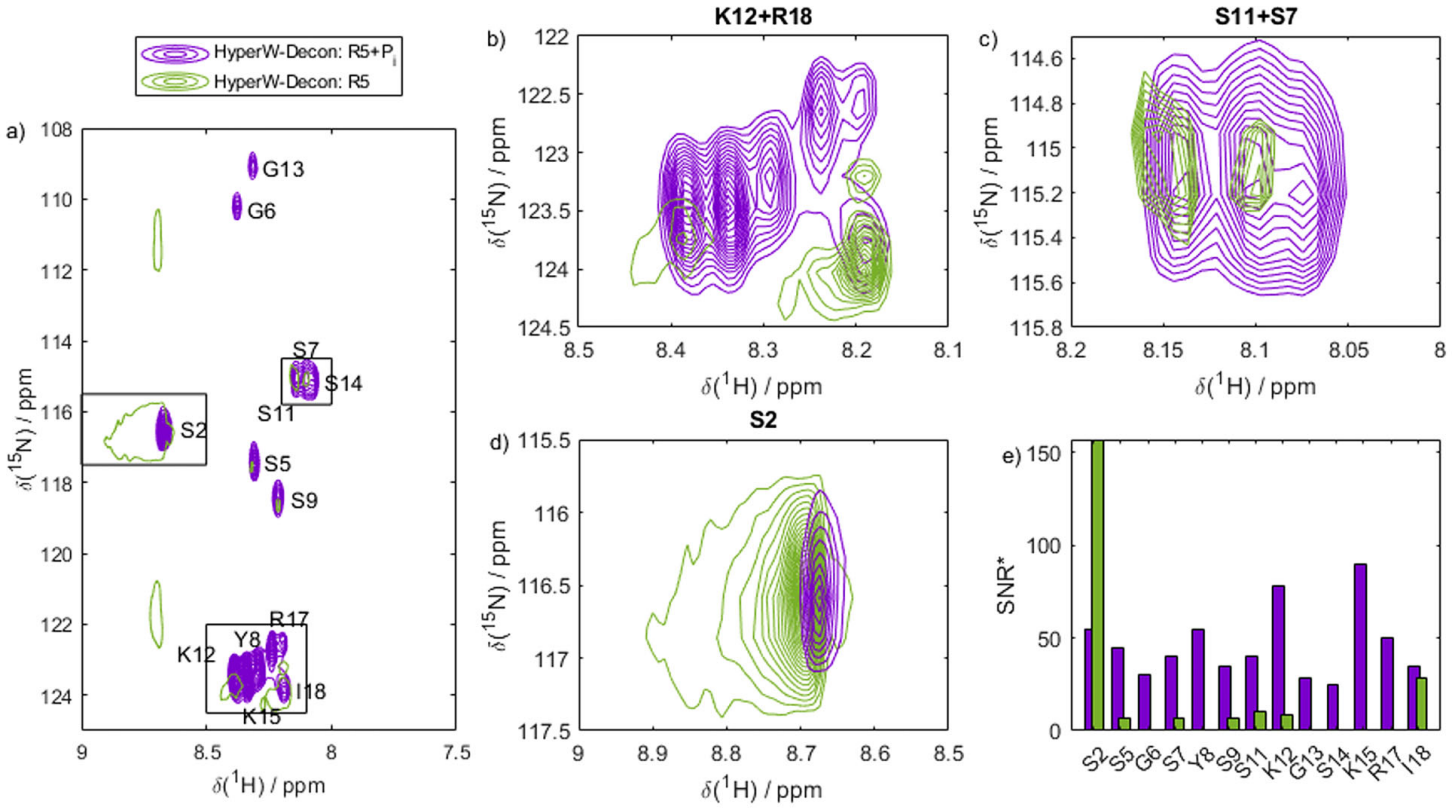
Monitoring R5‐P_i_ binding by HyperW within 60 s. a) HyperW deconvoluted spectra of R5 in the absence (green, single contour) and in the presence of P_i_ counterions (purple). Chemical shift changes and intensity changes can be observed as a result of P_i_ binding. Spectra were recorded immediately after mixing the IDP with counterions/HyperW. b‐d) Zoom on selected resonances showing that residue‐resolved chemical shifts can be followed with HyperW‐deconvolution. e) Residue‐resolved signal strength. In the presence of P_i_ (purple), the SNR* (*i.e*., SNR after the deconvolution procedure) is significantly higher for most residues than in the absence (green) of any multivalent counterions.

For R5, a similar response to counterion exposure was observed. While chemical shift changes were less pronounced than for OPN^82‐131^ (Figure 9a–d), the changes in SNR* followed a comparable trend. Prior to phosphate P_i_ complexation, signal enhancements were restricted to a small subset of resonances. Notably, residue S2 was strongly polarized before counterion exposure (Figure 9e, green bars). Upon binding P_i_, however, hyperpolarization was distributed almost uniformly across the entire peptide sequence (Figure 9e, purple bars), with all residues effectively receiving hyperpolarized protons from the solvent.

**Figure 10:**
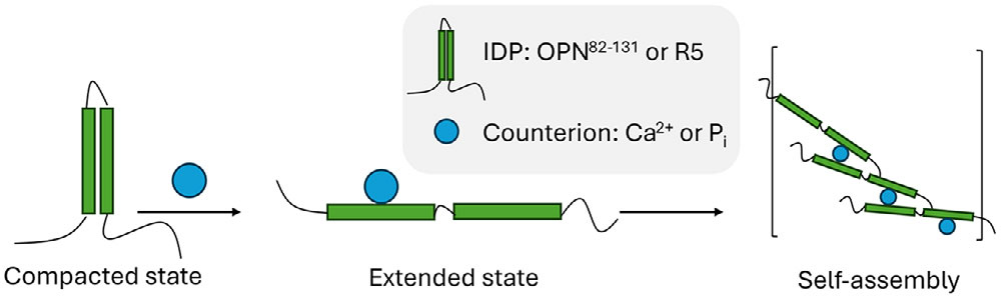
Coarse interaction model consistent with the observed counterion interactions. Both IDPs transiently adopt extended conformations before self‐assembly (simplified representation).

This observation agrees with recent reports^[^ ^60^ ^]^, which showed that in the absence of counterions, R5 adopts a transient β‐sheet conformation, that is, it features some β‐sheet propensities. This β‐sheet reduces solvent accessibility, limiting the efficiency of hyperpolarization transfer from HyperW. The observed small chemical shift changes also match expectations for a transition from a β‐sheet propensities into a fully unfolded state. This β‐sheet reduces solvent accessibility, limiting the efficiency of hyperpolarization transfer from HyperW. Binding P_i_ disrupts the β‐sheet, leading to an unfolded conformation that exposes the peptide backbone to the solvent, thereby enabling efficient hyperpolarization transfer and higher SNR*. These extended conformations then self‐assemble in a second step into larger structures as the intermolecular binding sites^[^ ^60^ ^]^ (residues 2–4 and 15–19) become exposed. These independent findings, thus, confirm and validate our results and data interpretation. Figure 10 sketches the identified conformational extension processes upon counterion encounter consistent with the herein reported observations.

Notably, the R5 signal intensities dropped rapidly within minutes after counterion exposure, reflecting the formation of NMR‐invisible self‐assemblies (see also ref. [60]). The observations in Figure 9 were made possible solely by our proposed methodology, highlighting HyperW‐Decon's ability to probe transient states rapidly, within seconds after formation, that would otherwise be inaccessible with conventional NMR.

To support this data interpretation, two alternative sources of the observed signals should be noted. (*i*) The NMR‐invisible self‐assemblies would also remain undetected by HyperW‐Decon due to strong line broadening in the direct‐detected (^1^H) dimension. Hence, rapid formation of self‐assembly followed by signal enhancement by HyperW can be ruled out.^[^ ^60^ ^]^ (*ii*) Residual monomers or oligomers in solution coexisting with the self‐assemblies show markedly different spectra than those seen by HyperW‐Decon.^[^ ^60^ ^]^ Also, this explanation can be ruled out.

Finally, it should be pointed out that while a comparison to state‐of‐the‐art spectra can be made for the noninteracting cases (Figures 4, 6, 7), this was not possible for the self‐assembly cases, as the assemblies and their precursors could not be tracked with established means. As shown in references [60, 62], for both peptides signals remaining after self‐assembly only stem from nonaggregated residual peptides in solution, impeding a comparison with the data shown in Figures 8 and 9. In contrast, the HyperW‐Decon approach was shown herein for two independent cases to be capable of accessing such formerly inaccessible states.

Due to the transient nature of the investigated encounters, which occur on the scale of seconds, conventional SOFAST‐ or BEST‐HMQC experiments lack the sensitivity as signal averaging becomes impossible. At the same time, the delay for sample and spectrometer preparation in traditional experiments might already exceed the lifetime of the observed intermediates. Increasing substrate concentrations and optimizing buffer conditions (e.g., deuteration) might help to boost signal intensities, but the experimental conditions still need to be optimized for each individual target. The proposed HyperW‐based approach might, thus, offer a broadly applicable and more accessible method.

## 3 Conclusions

Our study highlights the potential of hyperpolarized water (HyperW) combined with ML‐based deconvolution for addressing low sensitivity limitations in biomolecular NMR and characterizing transient biomolecular interactions with atomistic resolution. By compensating the inherent resolution challenges of dDNP, this approach might significantly expand the applicability of hyperpolarized NMR, including IDPs and other challenging systems, particularly those involving short‐lived intermediates. Across four distinct cases, we obtained spectra with resolution comparable to that of high‐field NMR, yet with sensitivity gains exceeding two orders of magnitude.

A key advancement is the ability to resolve short‐lived states of IDPs and peptides that are undetectable by conventional means. Using OPN and R5 as test cases, the HyperW methodology enabled the rapid acquisition of high‐resolution spectra within seconds of counterion interaction, revealing structural dynamics of early‐stage intermediates of biomineralization processes. Traditional NMR methods often fail to detect fleeting or low‐abundance species, creating gaps in our understanding of dynamic processes. HyperW, coupled with deconvolution, addresses this limitation by enhancing sensitivity while aiming not to compromise spectral resolution.

Importantly, the transient encounter states observed in this study align closely with prior molecular dynamics simulations and complementary experimental data^[^ ^14^, ^60^, ^71^, ^72^ ^]^, providing what is, to our knowledge, the first experimental validation of the two‐step interaction model for self‐assembling peptides proposed earlier^[^ ^60^ ^]^. The intermediate species, with lifetimes on the order of seconds, remained inaccessible by conventional NMR or biophysical techniques, underscoring the capability of HyperW‐Decon to resolve such short‐lived intermediates under near‐native conditions. Traditional techniques applied in our earlier work^[^ ^60^ ^]^, including modulation of kinetics *via* toggling concentrations, were not capable of detecting these intermediates. It should be noted, though, that approaches based on concentration or viscosity manipulation are limited, as they might perturb conditions critical to mineralization pathways when deviating significantly from native‐like environments. In contrast, our approach capitalizes on dDNP‐enhanced acquisition speed and sensitivity to capture these fleeting structural states with high resolution and under conditions that preserve native biochemical interactions. This capability supplements prior models by providing the missing experimental confirmation for mechanistic hypotheses that, until now, have relied solely on computational or indirect inference.

Notably, both IDPs exhibited similar counterion‐induced responses, characterized by increased solvent interactions associated with conformational expansion and solvent exposure of intermolecular interaction sites. Given the analogous nature of their respective biomineralization processes–where counterion‐induced self‐assembly precedes mineralization–these observations may reflect a shared mechanistic feature among peptide‐driven biomineralizing systems.

Beyond OPN and R5, the presented methodology offers a versatile framework for investigating other IDPs and peptides involved in biomolecular and biomimetic pathways. The ability to probe short‐lived conformational states can provide valuable insights into processes such as molecular recognition, enzymatic catalysis, or controlled release applications. Thus, the HyperW‐Decon approach might extend the utility of hyperpolarized NMR to new frontiers in structural biology, biophysics, and materials science. By extending hyperpolarization to previously inaccessible chemical systems, our work paves the way for applications in catalysis, materials science, and molecular dynamics investigation.

## Supporting Information

Peptide expression and purification, details on dDNP and NMR experiments, methodological considerations, signal assignments, and supplementary NMR data.

## Conflict of Interest

The authors declare no conflict of interest.

## Supporting information



Supporting Information
