# Supplementary material for: Deep Learning for Hyperpolarized NMR of Intrinsically Disordered Proteins without Resolution Loss: Access to Short‐Lived Intermediates
Source: Chemistry. 2025 Aug 8;31(59):e02067. doi: 10.1002/chem.202502067 (PMC12548504; doi:10.1002/chem.202502067)
Supplement: Supplementary file 1 — Supporting Information [file CHEM-31-e02067-s001.docx]

# Materials and Methods

## Sample Production

R5 was subcloned as a his-tagged SUMO-fusion construct into a pET-21a(+) expression vector and transformed into E. coli Rosetta2 cells. For protein expression, bacteria were grown at 37° C in LB media until transferred to M9 for ^13^C and/or ^15^N labeling (^13^C_6_ glucose and/or ^15^N ammonium chloride added at 1 g/L). Cells were induced with isopropyl-β-ᴅ-thiogalactopyranoside at an optical density corresponding to A600 = 0.6 and incubated at 30° C overnight. Cells were homogenized in a solution of TRIS (25 mM), NaCl (100 mM), and β-mercaptoethanol (2 mM) at pH 8. The resulting supernatant was purified by Ni^2+^-affinity chromatography, and fractions pooled when a mass of 15.470 kDa (^15^N labeled) and 16.449 kDa (^13^C/^15^N labeled), were found in LC-MS analyses. Cleavage of His and SUMO (small ubiquitin-related modifier)-tag was achieved by SUMO protease digestion overnight, confirmation was done by mass spectrometry, reporting masses of 13.448 kDa of His-SUMO-tag (^15^N labeled), and 14.355 kDa of His-SUMO-tag (^13^C/^15^N labeled). The cleaved peptide mixture was purified by Kromasyl C4 semi-preparative RP-HPLC column on a Waters Prep 150 System using a gradient from 5 % - 65 % of water/acetonitrile (0.08% v/v) in water/trifluoroacetic acid (0.01% v/v) over 30 minutes at a flow rate of 5 mL/min. The fractions with UV absorption above 70 mAu were collected automatically. For analysis, 15 μL of each fraction were directly injected into a Thermo Fisher HPLC-MS system to identify product-containing fractions. Fractions were finally pooled accordingly and lyophilized.

Osteopontin^82-131^ (Uniprot entry P10451 · OSTP_HUMAN) was subcloned as a his-tagged SUMO-fusion with a TEV cleavage site construct into a pET-21a(+) expression vector and transformed into E. coli Rosetta2 cells. For protein expression, bacteria were grown at 37° C in LB media until transferred to M9 for ^13^C and/or ^15^N labeling (^13^C_6_ glucose and/or ^15^N ammonium chloride added at 1 g/L). Cells were induced with isopropyl-β-ᴅ-thiogalactopyranoside at an optical density corresponding to A600 = 0.7 and incubated at 30° C overnight. Cells were homogenized in a solution of TRIS (25 mM), NaCl (100 mM), and β-mercaptoethanol (2 mM) at pH 8. The resulting supernatant was purified by Ni^2+^-affinity chromatography, and fractions pooled when a mass of 22.2 kDa (^13^C/^15^N labeled), were found in LC-MS analyses. Cleavage of His and SUMO (small ubiquitin-related modifier)-tag was achieved by TEV protease digestion overnight, and confirmed by mass spectrometry, reporting masses of 6.18 kDa of Osteopontin (^13^C/^15^N labeled). The cleaved peptide mixture was buffer exchanged into a solution of TRIS (25 mM), NaCl (100 mM), and β-mercaptoethanol (2 mM) at pH 8 and purified again by Ni^2+^-affinity chromatography. The flow-through was collected in fractions. To identify product-containing fractions, LC-MS analyses were performed. Osteopontin-containing fractions were finally pooled and lyophilized.

## NMR Spectroscopy

The signal assignment of the R5 backbone resonances using a combination of HNCO, HNCACB, HNN, and HN(C)N experiments was previously reported in reference^1^. Signal assignment of Osteopontin^H82-E131^ was achieved using the HNN pulse sequence^2^ . Spectral widths for F1 and F2 were 709.792 Hz and 10 ppm and 9803.922 Hz and 13.9975 ppm for F3. In both indirect dimensions, the HNN was recorded in States-TPPI mode for quadrature detection using 40 and 60 increments, respectively, with offsets of 3291.88 and 4.700 ppm for ^1^H and 8516.51 Hz and 120 ppm for ^15^N. Detection was carried out using a 700 MHz Bruker HDIII spectrometer equipped with a QCIF helium-cooled cryogenic probe at 25 °C.

## Dissolution DNP

For DNP 200 μL of a solution of 15 mM TEMPOL in a mixture of 15% or 30% glycerol-d_8_ and 85% or 70% H_2_O (see below) were hyperpolarized at a temperature of 1.4 K in a magnetic field of 6.7 T for 5000 s using continuous-wave microwave irradiation at 188 GHz. DNP samples were always freshly prepared to avoid ripening effects.^3^ A VDI microwave source was used together with a 16x frequency multiplier that provided an output power for the microwave of ca. 50 mW. The magnet-cryostat combination was purchased from Cryogenic Ltd. and operated as described in reference ^4^.

For detection of the proton solid-state polarization, a 400 MHz Bruker NEO system was adapted to a ^1^H resonance frequency of 285.3 MHz. The detection circuit and the external tune-and-match system were home-built, as described in reference ^5^. To monitor the build-up, detection pulses with a flip angle of 1 degree were applied every 5 s.

After DNP, the sample was dissolved with a burst of 5 mL D_2_O at 1.5 MPa as described in reference ^4^. The hyperpolarized liquid was then pushed with helium gas at 0.7 MPa to the detection spectrometer. The dissolution process employed a home-built pressure heater actuated with an Arduino microcontroller. Injection of a predefined hyperpolarized solution (300 µL) into a Shigemi NMR tube (Shigemi Ltd.) was performed through a prototype with a second Arduino microcontroller within 2 s. ^6^

Detection in the liquid state was carried out using a 700 MHz Bruker HDIII spectrometer equipped with a QCIF helium-cooled cryogenic probe. The pulse sequence for detection corresponded to a BEST-HMQC ^7-9^. We used PC9 and RSNOB^10^ selective 90° and 180° pulses covering a bandwidth of 4 ppm centered around a carrier frequency of 9.0 ppm to excite and invert the protons (not a 120° pulse as the polarization recovery mechanism is different from thermal equilibrium NMR). The pulse lengths were 2.06 ms and 686 µsec, respectively. The 90° pulse for the ^15^N channel was 37 µsec long. The ^15^N carrier frequency was adjusted to 117 ppm. Heteronuclear decoupling was achieved using the GARP^11^ scheme as preinstalled in Bruker TOPSPIN 4. The recycling delay d1 was set to 0.2 s, the acquisition time was 0.1 s leading to a total recycling time of 0.3 s. In total, 64 *t*_1_ increments were recorded with a step length of 0.2 ms.

The protein solutions for dDNP contained 2 mg/mL of protein before three-fold dilution with hyperpolarized water, as described in the main text. Both peptide R5 and OPN were provided in 150 μL buffered solution at pH 6.5 or 3, respectively. After dilution, the sample volume was 450 μL, and the peptide concentration was consequently 2/3 mg/mL.

Reference spectra were recorded with the same sample concentration but with 512 *t*_1_-increments instead of 64 and 128 averages per FID instead of 2.

Multiple HyperW experiments were carried out with the two-fold objective of testing how the deconvolution approach would fare against conventional methods and whether it would allow us to investigate the sample behavior further than was previously possible. For OPN^82-131^, two different conditions were studied.

1. **Osteopontin mixed with hyperpolarized water:**

For the DNP, a mixture of 200 μL of a solution of 15 mM TEMPOL ,15% glycerol-d_8_ (30 µL, 41 mg) and 85% H_2_O (170 µL) was hyperpolarized as described before. OPN^82-131^ at a concentration of 2 mg/mL (150 µL) buffered to pH 6.5 in 0.05 M HEPES was prefilled into a Shigemi tube and mixed with 300 µL of dissolved hyperpolarized water.

1. **Osteopontin mixed with hyperpolarized water containing calcium ions:**

DNP experiments were performed as described in point 1 with the addition of 0.5 M CaCl_2_ (11 mg) to the DNP mixture. The final concentration of calcium ions upon injection and mixing was, consequently, 12.8 mM.

Two experiments were carried out to investigate R5 under similar conditions.

1. **R5 mixed with hyperpolarized water:**

For DNP, 200 μL of a solution of 15 mM TEMPOL in a mixture of 30% glycerol-d_8_ (60 µL, 82 mg) and 70% H_2_O (140 µL) were hyperpolarized. The mixing experiment was performed exactly as described above.

1. **R5 mixed with hyperpolarized water containing phosphate ions:**

DNP experiments were performed as in point 1 with the addition of 0.5M K_2_HPO_4_ (17.4 mg) to the DNP mixture. The final concentration of phosphate ions upon injection was consquently12.8 mM.

Note that all samples were prepared with D_2_O instead of H_2_O, if not otherwise explicitly stated. The total dolution of the HyperW resulted in a H_2_O content of 2% after completion of the mixing process. Furthermore, the buffer strength was sufficient in all experiments such that the pH values did not change during the dDNP experiments.

Signal enhancements were calculated as either ε = SNR_hyp_/ SNR_ref_ * RG_ref_/RG_hyp_ * NS_ref_/NS_hyp_ or as ε* = SNR_hyp_/ SNR_ref_. For these calculations the same sample was used to measure the reference and the hyperpolarized spectrum.

## Machine Learning

The neural network was trained to predict exponential decay rates from simulated signals derived from processed experimental data. First, the raw experimental spectra were subjected to an inverse Fourier transform to convert them into time-domain signals. These signals were then modulated with a weighting function to simulate exponentially decaying signals. The decay was modelled as the *E*(*t*) function described in the main text plus different noise levels, where λ is the decay rate. Each signal was normalized to its initial value and scaled with a random factor to enhance variability, reflecting different signal enhancements. The processed signals were stored in a dataset, which included 100000 samples per combination of decay rates and free induction decays (FIDs). The true decay rates (λ) were evenly spaced and assigned to the corresponding signals for supervised training.

A fully connected (fc) feedforward neural network was constructed to predict the decay rates. The network consisted of an input layer accepting 512-dimensional feature vectors, followed by three hidden layers with 512, 256, and 128 neurons, respectively. Each hidden layer used rectified linear unit (ReLU) activation functions, and dropout layers with a rate of 0.3 were included after the first two hidden layers to mitigate overfitting. The output layer contained a single neuron for predicting the decay rate as a continuous value, and the model was trained using a regression loss function.

The dataset was randomly divided into training and testing sets using an 80:20 split. The data splitting procedure has been chosen to avoid data leakage (training set generated separately from the validation data and scaled within the simulation process). To improve robustness and generalization, Gaussian noise (σ=0.05) was added to the input signals. The training process employed the Adam optimizer with an initial learning rate of 0.001, a batch size of 64, and a total of 100 epochs. The validation set was used to monitor the model's performance during training.

The loss function was mean squared error (MSE), and gradient updates were performed using backpropagation. Early stopping was not applied, but validation was conducted at fixed intervals using the held-out test set. To monitor model convergence, training progress was recorded, including loss curves for both the training and validation sets.

After training, the model’s performance was evaluated on the test set. Predictions for unseen signals were compared to the true decay rates, and metrics such as mean squared error (MSE) and mean absolute error (MAE) were computed. For the DNN trained on R5, the MSE was 0.2574 and the MAE was 0.3672. For the DNN resulting from training on OPN, the MSE was 0.2200 and the MAE was 0.4690. Additionally, the correlation between true and predicted decay rates was analyzed to assess the model’s accuracy. This approach enabled the network to effectively predict decay rates from noisy, simulated signals, as shown in the main text. Figure S1a shows a sketch of the generated network. Figures S1b and S1c show the correlation between the true and predicted decay rates of R5 and OPN, respectively.

To further evaluate the contribution of different model components, ablation studies were conducted by systematically removing or modifying network elements and analyzing the resulting impact on performance. The first ablation experiment removed dropout layers to assess the role of regularization in preventing overfitting. A second study investigated the effect of reducing the network depth by removing the second hidden layer, determining whether a simpler architecture could maintain predictive accuracy. Additionally, the effect of the input signal length was tested by training a variant of the model using only the first 32 time points instead of the full 64. The sensitivity of the network to noise augmentation was assessed by training a model without added noise and comparing its performance to the baseline. In all ablation studies, the modified models were retrained under identical conditions, and their MSE and MAE on the test set were compared to the original architecture. These tests provided insight into the necessity of each component and the robustness of the model under different conditions.


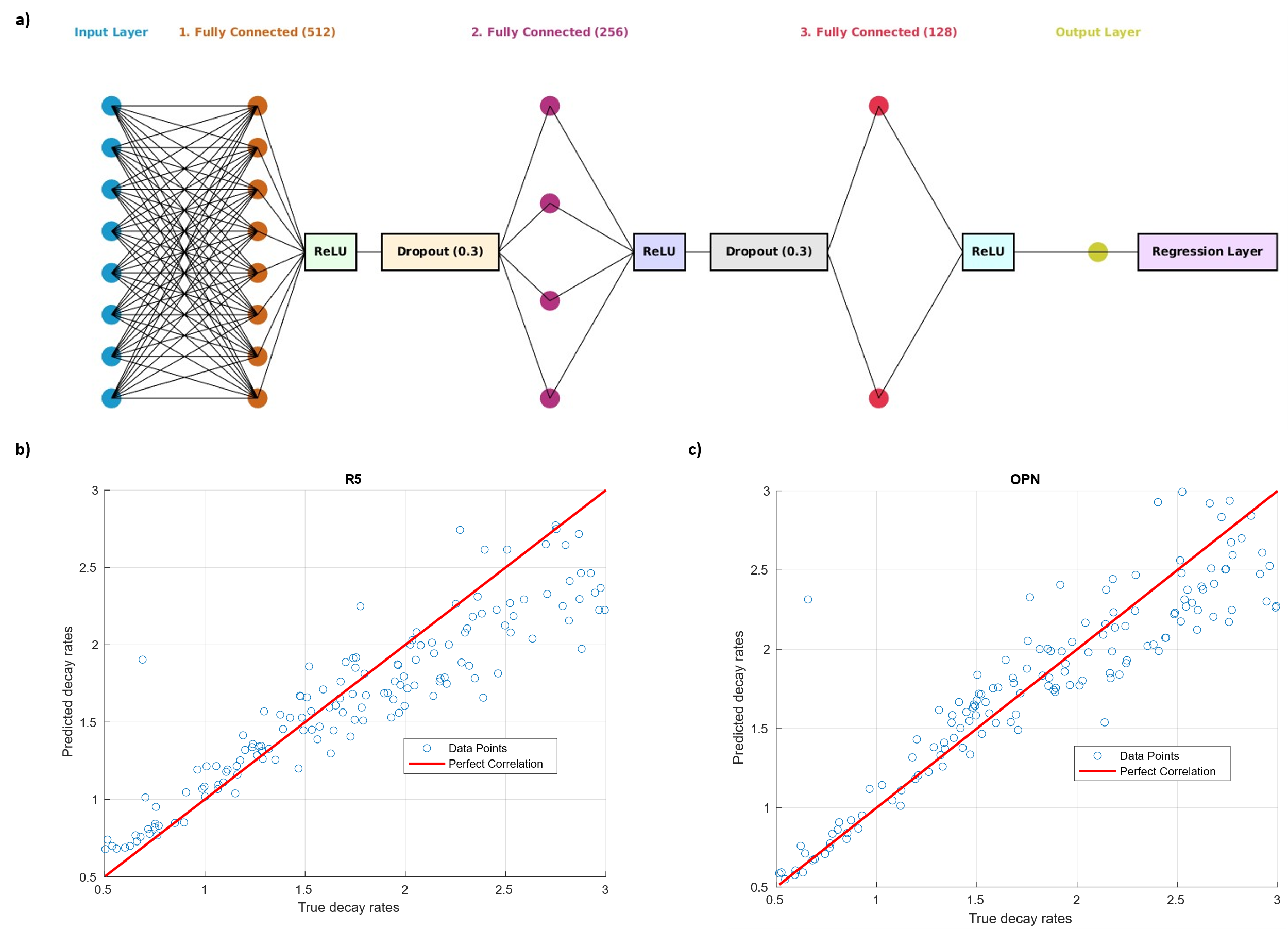


**Figure S1. a)** Simplified representation of the architecture of the deep neural networks (DNNs) used for hyperpolarised water deconvolution. **b)** Comparison of the predicted decay rates by the DNN trained on R5 with the true decay rates. **c)** Comparison of the predicted decay rates by the DNN trained on OPN with the true decay rates.

The corresponding MATLAB code can be found below:

% This script imports data processed by Bruker TopSpin (2rr files) and usesit to gernete training data for machine leatning.

% For the training a thermal equilibrium spectrum is used. Then an

% indepdendent hyperpolarized spectrum is analyzed with the trained data.

% Code was run and tasted with MATALB 2022b and it took ca. 30 min on a PC equipped with an I5

% 13000-600 KF and a GeForce RTX 3060.

% Clear workspace

clear; clc;

%Windows

brukerData1 = brukerimport(1, 'datapath'); % here we use GNAT to load the data for training (https://www.nmr.chemistry.manchester.ac.uk/?q=node/430)

%Linux

%brukerData1 = brukerimport(1, 'datapath'); % path/to/your/file

brukerSpec1 = brukerData1.X2DSPEC'; % this is the complex sepctrum in numerical format

% create synthetic training data

num_of_exps = 2000;

true_exps = linspace(0.4,4,num_of_exps);

num_of_FIDs = 50; % change to number of bins of the interferogram along f2 to be tested

data_ifft2 = ifft(brukerSpec1);

sdat = size(data_ifft2);

data_ifft = real(data_ifft2(1:sdat(1)/2,:));

data_APOD_store = zeros(64,num_of_exps.*num_of_FIDs);

idx = reshape(501:600,[],num_of_FIDs); % here select region of the interferogram with the data

for j = 1:num_of_FIDs

data_idx = data_ifft(:,idx(:,j));

for i = 1:num_of_exps

w = exp((-true_exps(i)).*(0:sdat(1)/2-1))'+(1+randn(1))./50; % exogeneous decay function

data = (sum(data_idx,2));

data = data./data(1);

data_APOD = data.*w.*(100+50*randn(1));

data_APOD_store(:,(j-1)*num_of_exps+i) = data_APOD ;

end

end

data = data_APOD_store; % Extract the dataset

[num_samples, num_time_points, num_classes] = size(data);

% Define the true values of the second exponential

true_values = repmat(true_exps',num_of_FIDs,1);

% Prepare the data

X = data_APOD_store'; % Transpose to make each row an oscillation (101 x 64)

y = true_values(1:size(X, 1)); % Corresponding true values

% Add noise to the dataset for robustness

noise_variation = 0.05 * randn(size(X));

X_noisy = X + noise_variation;

% Split the data into training and testing sets

cv = cvpartition(size(X_noisy, 1), 'HoldOut', 0.2); % 80-20 train-test split

trainIdx = training(cv);

testIdx = test(cv);

if max(trainIdx) > size(X_noisy, 1) || max(testIdx) > size(X_noisy, 1)

error('Index out of bounds for the dataset.');

end

X_train = X_noisy(training(cv), :);

y_train = y(training(cv), :);

X_test = X_noisy(test(cv), :);

y_test = y(test(cv), :);

% Define neural network architecture

layers = [

featureInputLayer(size(X_train, 2)) % Input layer

fullyConnectedLayer(512) % Hidden layer with 512 neurons

reluLayer % Activation function

dropoutLayer(0.3) % Dropout for regularization

fullyConnectedLayer(256) % Hidden layer with 256 neurons

reluLayer

dropoutLayer(0.3)

fullyConnectedLayer(128) % Hidden layer with 128 neurons

reluLayer

fullyConnectedLayer(1) % Output layer

regressionLayer % Regression output layer

];

% Visualize the network architecture

%analyzeNetwork(layers);

% Training options

options = trainingOptions('adam', ...

'MaxEpochs', 100, ...

'MiniBatchSize', 64, ...

'InitialLearnRate', 0.001, ...

'ValidationData', {X_test, y_test}, ...

'ValidationFrequency', 10, ...

'Plots', 'training-progress', ...

'Verbose', false);

% Train the neural network

net = trainNetwork(X_train, y_train, layers, options);

% Test the network

y_pred = predict(net, X_test);

% Evaluate performance

test_loss = mean((y_test - y_pred).^2); % Mean Squared Error

test_mae = mean(abs(y_test - y_pred)); % Mean Absolute Error

% Display results

fprintf('Test Loss (MSE): %.4f\n', test_loss);

fprintf('Test MAE: %.4f\n', test_mae);

% Plot correlation

figure;

scatter(y_test, y_pred, 'o', 'DisplayName', 'Data Points');

hold on;

plot([min(y_test), max(y_test)], [min(y_test), max(y_test)], 'r-', ...

'LineWidth', 2, 'DisplayName', 'Perfect Correlation');

xlabel('True Values');

ylabel('Predicted Values');

axis([min(true_exps) max(true_exps) min(true_exps) max(true_exps)]);

legend('Location', 'best');

grid on; title('correlation plot');

%% Here the actual HyperW data is analyzed

%Windows

brukerData2 = brukerimport(1, '2\pdata\1'); % path\to\your\file

%Linux

%brukerData2 = brukerimport(1, '2/pdata/1'); % path/to/your/file

brukerSpec2 = brukerData2.X2DSPEC';

data_ifft2 = ifft(brukerSpec2);

sdat = size(data_ifft2);

data_ifft2 = real(data_ifft2(1:sdat(1)/2,:));

idx = reshape(501:600,[],num_of_FIDs);

for j = 1:num_of_FIDs % here I look at various fits

data_idx = data_ifft2(:,idx(:,j)');

data = (sum(data_idx,2));

data = data./data(1);

decay_rate(j) = predict(net,2.*data');

figure(11);

subplot(ceil(sqrt(num_of_FIDs)),ceil(sqrt(num_of_FIDs)),j);

fitfun = exp(-decay_rate(j).*(1:64));

plot(1:64,data,1:64,fitfun./max(fitfun)); title(num2str(round(decay_rate(j),3)));

end

figure(202); title('distribution of decay rates')

histfit(decay_rate.*0.4); xlabel('R_{1,eff} / ms'); ylabel('N'); axis tight; %Note: values corrected for actual t1 increment

## Methodological Considerations

Several methodological considerations need to be taken into account, when applying the HyperW-Decon approach:

1. The assumption of a single, uniform decay constant *R* is broadly valid under typical dDNP conditions, where the hyperpolarized water rapidly and homogeneously mixes with the sample. In IDPs (such as the herein studies systems), exchange between HyperW and peptide is typically fast and does not vary so strongly between different residues.^12, 13^ The assumption may fail in heterogeneous (e.g., phase-separated systems or compartmentalized organelles) or folded proteins with distinct and shielded compartments exhibiting substantially different water interaction dynamics. In such a case, different exogenic decay rates might need to be taken into account to deconvolute each resonance’s FID correctly from the HyperW contribution.
2. We also tested least-square fitting methods due to the mathematically straightforward problem solution (eq. 1-3) However, unlike regression-based approaches, which estimate parameters solely by minimizing mathematical error between model and data, our DNN methodology is designed with a different objective: to find the correct HyperW-decay rate for a wide set of FIDs under changing conditions. While regression and least-squares fitting methods are mathematically well-defined, we found them less suited for dDNP-derived data.
3. First, linear regression and least-squares methods were found to be more affected by noise in our hands, which is variable and resonance-specific in dDNP experiments. Minor fluctuations in baseline or acquisition artefacts can bias the fitted decay constant, resulting in under- or over-correction during deconvolution. Second, these approaches were found to be strongly affected by intrinsic heterogeneity in FID decay rates due to varying relaxation behaviors or overlapping peaks. Third, we observed that these analytical methods often led to artificial narrowing or broadening of line shapes, introducing systematic distortions that degrade spectral interpretability rather than enhance it.
4. Moreover, simpler heuristic approaches—such as manually tuning *R*_1,train_ via trial-and-error to optimize apparent resolution—lack reproducibility and introduce subjective bias. They offer no quantitative criterion to assess whether a spectrum has been corrected appropriately, nor can they be generalized across different experimental conditions.
5. In contrast, our DNN is trained end-to-end on large datasets of known spectra with corresponding distorted FIDs, learning a nonlinear mapping from input to optimal correction function. Crucially, this training is implicitly guided by a global objective: maximizing the fidelity of the reconstructed spectrum to its known artefact-free counterpart. The network thus learns to recognize and suppress noise-induced deviations, correct for heterogeneous decay patterns, and restore native spectral features, without ever requiring the intrinsic FID to be explicitly known or modeled.

Our approach proved robust across all tested datasets, consistently yielding high-quality spectra with native-like linewidths, regardless of the FID’s noise profile or degree of truncation. Its capacity to generalize across diverse conditions and proteins, while maintaining spectral integrity, establishes the DNN method as not just a more automated alternative, but a categorical solution to traditional fitting schemes in the context of dDNP-enhanced NMR.

1. Further, we note that in processing 2D NMR spectra, especially under the constraints of dDNP experiments, truncation of the indirect dimension (t₁) is often unavoidable due to the limited acquisition time dictated by hyperpolarization decay.^14^ This leads to characteristic sinc-type artefacts and reduced resolution in the frequency domain. Apodization—often implemented via sine-bell, exponential, or Gaussian functions—serves to mitigate truncation effects by dampening the tail of the FID, thereby reducing spectral leakage and improving peak shape.^15^ As the acquisition time in the indirect dimension is limited in a HyperW experiment, apodization of truncated FIDs might additionally introduce line broadening. Our approach corrects for global decay distortions induced by the hyperpolarization, but does not interfere with the apodization of the intrinsic FID of the detected resonance, allowing the preservation of the linewidth that would be obtained with a similar detection scheme, but in the absence of any hyperpolarization. Thus, our deep learning–based correction achieves resolution enhancement, while truncation artifacts can be handled via conventional data treatment methods.

## Signal Assignments

Table S1. OPN^82-131^ resonance assignments.

| **δ(^15^N) / ppm** | **δ(^1^H) / ppm** | **Residue Number** | **δ(^15^N) / ppm** | **δ(^1^H) / ppm** | **Residue Number** |
| --- | --- | --- | --- | --- | --- |
| 121 | 8.505 | **H82** | 116.9 | 8.404 | **S120** |
| 121.5 | 8.363 | **D83** | 122.5 | 8.365 | **D121** |
| 120.7 | 8.333 | **H84** | 121.5 | 8.4 | **E122** |
| 116.4 | 8.3 | **M85** | 116.3 | 8.26 | **S123** |
| 122.7 | 8.352 | **D86** | 120.2 | 8.313 | **H124** |
| 120.9 | 8.215 | **D87** | 120.8 | 8.463 | **H125** |
| 117 | 7.994 | **M88** | 118.1 | 8.463 | **S126** |
| 120.9 | 8.543 | **D89** | 122.7 | 8.506 | **D127** |
| 117.9 | 8.453 | **D90** | 120.7 | 8.329 | **E128** |
| 121.4 | 8.452 | **E91** | 116.3 | 8.285 | **S129** |
| 120.3 | 8.353 | **D92** | 123.2 | 8.373 | **D130** |
| 119.2 | 8.205 | **D93** | 115.4 | 7.885 | **E131** |
| 119.5 | 8.164 | **D94** |  |  |  |
| 121.4 | 8.321 | **D95** |  |  |  |
| 115.9 | 8.205 | **H96** |  |  |  |
| 122.4 | 8.131 | **V97** |  |  |  |
| 124.5 | 8.382 | **D98** |  |  |  |
| 116.7 | 8.241 | **S99** |  |  |  |
| 121.1 | 8.285 | **Q100** |  |  |  |
| 120.4 | 8.242 | **D101** |  |  |  |
| 118.7 | 8.297 | **S102** |  |  |  |
| 121.8 | 8.126 | **I103** |  |  |  |
| 124.6 | 8.509 | **D104** |  |  |  |
| 116.7 | 8.283 | **S105** |  |  |  |
| 122.2 | 8.472 | **N106** |  |  |  |
| 121.3 | 8.305 | **D107** |  |  |  |
| 115.8 | 8.206 | **S108** |  |  |  |
| 122.6 | 8.352 | **D109** |  |  |  |
| 120.9 | 8.247 | **D110** |  |  |  |
| 119.9 | 8.017 | **V111** |  |  |  |
| 124.2 | 8.396 | **D112** |  |  |  |
| 122.3 | 8.335 | **D113** |  |  |  |
| 124.1 | 8.165 | **T114** |  |  |  |
| 122.7 | 8.33 | **D115** |  |  |  |
| 122 | 8.314 | **D116** |  |  |  |
| 116 | 8.265 | **S117** |  |  |  |
| 119.9 | 8.351 | **H118** |  |  |  |
| 121.2 | 8.277 | **Q119** |  |  |  |
| **δ(^15^N) / ppm** | **δ(^1^H) / ppm** | **Residue Number** | **δ(^15^N) / ppm** | **δ(^1^H) / ppm** | **Residue Number** |
| 121 | 8.505 | **H82** | 116.9 | 8.404 | **S120** |
| 121.5 | 8.363 | **D83** | 122.5 | 8.365 | **D121** |
| 120.7 | 8.333 | **H84** | 121.5 | 8.4 | **E122** |
| 116.4 | 8.3 | **M85** | 116.3 | 8.26 | **S123** |
| 122.7 | 8.352 | **D86** | 120.2 | 8.313 | **H124** |
| 120.9 | 8.215 | **D87** | 120.8 | 8.463 | **H125** |
| 117 | 7.994 | **M88** | 118.1 | 8.463 | **S126** |
| 120.9 | 8.543 | **D89** | 122.7 | 8.506 | **D127** |
| 117.9 | 8.453 | **D90** | 120.7 | 8.329 | **E128** |
| 121.4 | 8.452 | **E91** | 116.3 | 8.285 | **S129** |
| 120.3 | 8.353 | **D92** | 123.2 | 8.373 | **D130** |
| 119.2 | 8.205 | **D93** | 115.4 | 7.885 | **E131** |
| 119.5 | 8.164 | **D94** |  |  |  |
| 121.4 | 8.321 | **D95** |  |  |  |
| 115.9 | 8.205 | **H96** |  |  |  |
| 122.4 | 8.131 | **V97** |  |  |  |
| 124.5 | 8.382 | **D98** |  |  |  |
| 116.7 | 8.241 | **S99** |  |  |  |
| 121.1 | 8.285 | **Q100** |  |  |  |
| 120.4 | 8.242 | **D101** |  |  |  |
| 118.7 | 8.297 | **S102** |  |  |  |
| 121.8 | 8.126 | **I103** |  |  |  |
| 124.6 | 8.509 | **D104** |  |  |  |
| 116.7 | 8.283 | **S105** |  |  |  |
| 122.2 | 8.472 | **N106** |  |  |  |
| 121.3 | 8.305 | **D107** |  |  |  |
| 115.8 | 8.206 | **S108** |  |  |  |
| 122.6 | 8.352 | **D109** |  |  |  |
| 120.9 | 8.247 | **D110** |  |  |  |
| 119.9 | 8.017 | **V111** |  |  |  |
| 124.2 | 8.396 | **D112** |  |  |  |
| 122.3 | 8.335 | **D113** |  |  |  |
| 124.1 | 8.165 | **T114** |  |  |  |
| 122.7 | 8.33 | **D115** |  |  |  |
| 122 | 8.314 | **D116** |  |  |  |
| 116 | 8.265 | **S117** |  |  |  |
| 119.9 | 8.351 | **H118** |  |  |  |
| 121.2 | 8.277 | **Q119** |  |  |  |

Table S2. R5 resonance assignments.

| **δ(^15^N) / ppm** | **δ(^1^H) / ppm** | **Residue Number** |
| --- | --- | --- |
| 117.024 | 8.690 | **S2** |
| 123.797 | 8.419 | **K3** |
| 123.303 | 8.469 | **K4** |
| 117.818 | 8.396 | **S5** |
| 110.941 | 8.459 | **G6** |
| 115.608 | 8.189 | **S7** |
| 122.759 | 8.327 | **Y8** |
| 118.777 | 8.299 | **S9** |
| 110.433 | 7.822 | **G10** |
| 115.57 | 8.227 | **S11** |
| 123.485 | 8.419 | **K12** |
| 109.831 | 8.397 | **G13** |
| 115.621 | 8.164 | **S14** |
| 123.335 | 8.379 | **K15** |
| 123.849 | 8.469 | **R16** |
| 122.629 | 8.275 | **R17** |
| 123.918 | 8.267 | **I18** |
| 130.808 | 8.056 | **L19** |

## Supplementary NMR Spectra

##
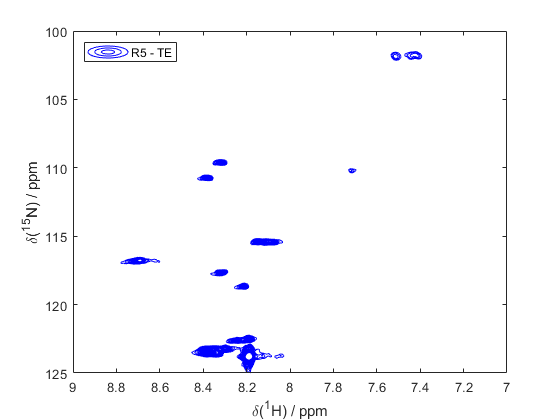


**A**


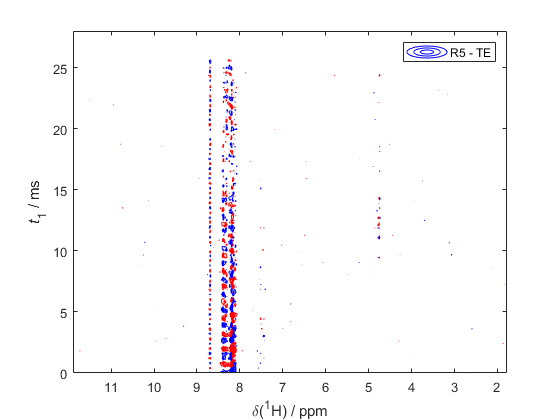


**B**

**Figure S2.** ^1^H-^15^N HMQC of R5 in thermal equilibrium (A) and corresponding interferogram (B).


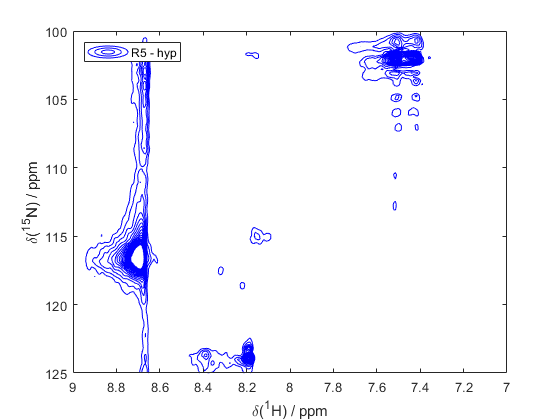


**A**


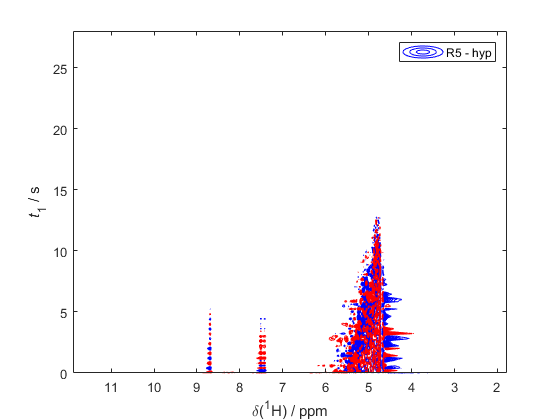


**B**

**Figure S3.** ^1^H-^15^N HMQC of R5 in HyperW (A) and corresponding interferogram (B).


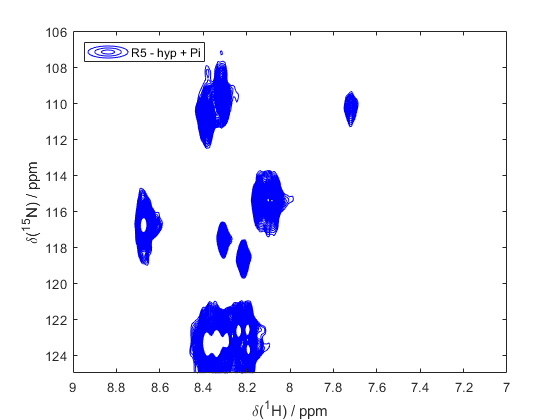


**A**


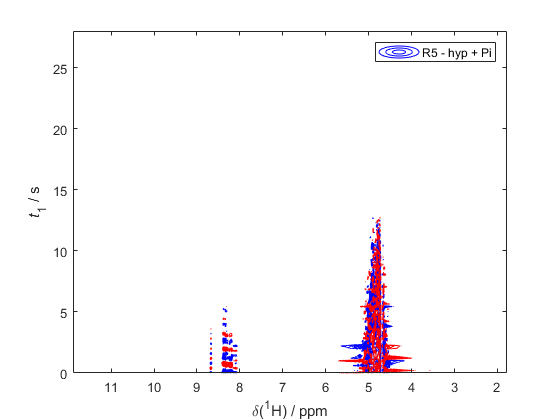


**B**

**Figure S4.** ^1^H-^15^N HMQC of R5 in HyperW and in the presence of phosphate (A) and corresponding interferogram (B).


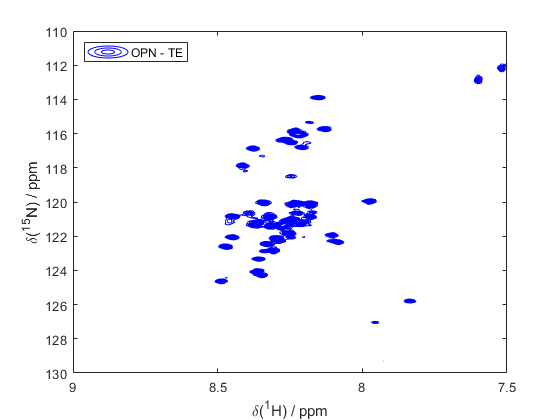


**A**


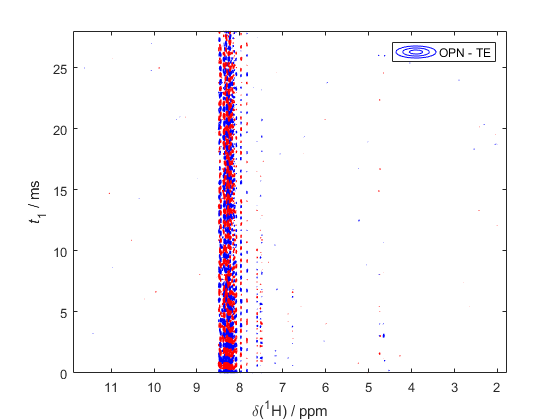


**B**

**Figure S5.** ^1^H-^15^N HMQC of OPN^82-131^ in thermal equilibrium (A) and corresponding interferogram (B).


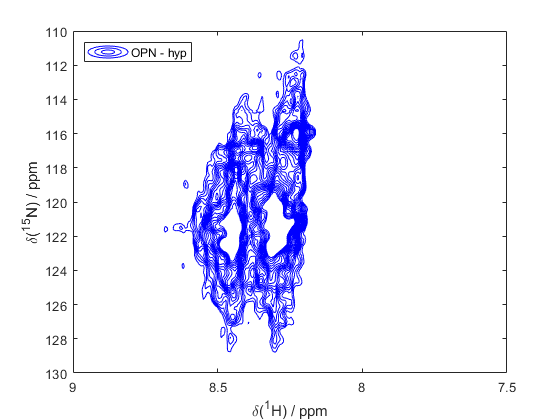


**A**


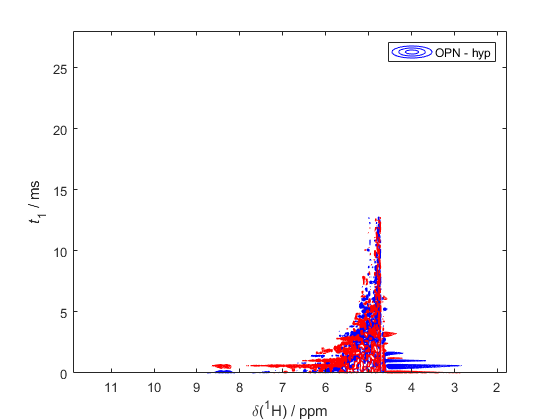


**B**

**Figure S6.** ^1^H-^15^N HMQC of OPN^82-131^ in HyperW (A) and corresponding interferogram (B).


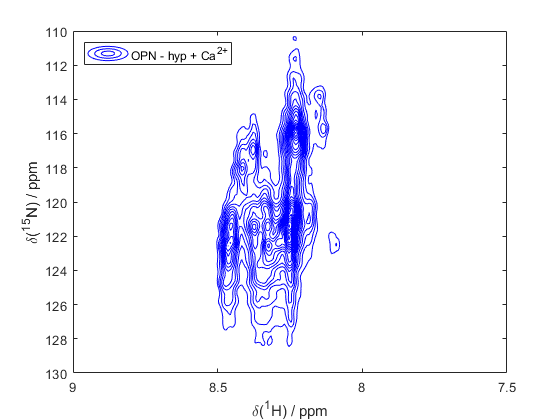


**A**


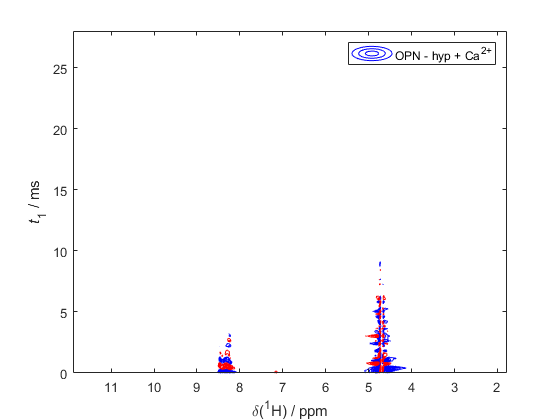


**B**

**Figure S7.** ^1^H-^15^N HMQC of OPN^82-131^ in HyperW and in the presence of Ca^2+^ (A) and corresponding interferogram (B).


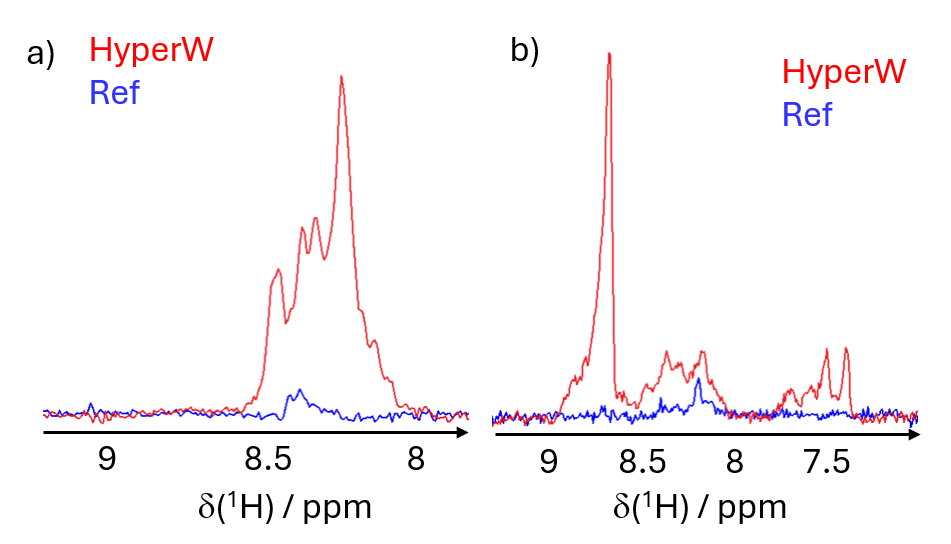


**Figure S8**. Comparison of the first increments of the hyperpolarized spectra (red) of R5 (rifht) and OPN (left), compared the same increment of the reference recorded with the exact same DNP sample and the same number of scans, but after decay of the hyperpolarization. The bulk enhancement on these first increments was 333 and 280, respectively.

**Figure S9.** Line shapes between HyperW (blue) and conventional (red) NMR taken from the spectra in Fig. 2b. The signals (except the noise) align quantitatively showing that no temperature drift is present.

## Supporting Information References

1. Kozak, F.; Brandis, D.; Pötzl, C.; Epasto, L. M.; Reichinger, D.; Obrist, D.; Peterlik, H.; Polyansky, A.; Zagrovic, B.; Daus, F.; Geyer, A.; Becker, C. F.; Kurzbach, D., An Atomistic View on the Mechanism of Diatom Peptide-Guided Biomimetic Silica Formation. *Advanced Science* **2024,** *11* (30), 2401239.

2. Panchal, S.; Bahavesh, N.; Hosur, R., Improved 3D triple resonance experiments, HNN and HN(C)N, for HNand 15N sequential correlations in (13C, 15N) labeled proteins: Application to unfolded proteins. *Journal of Biomolecular NMR* **2001,** *20*, 135-147.

3. Weber, E. M. M.; Sicoli, G.; Vezin, H.; Frébourg, G.; Abergel, D.; Bodenhausen, G.; Kurzbach, D., Sample Ripening through Nanophase Separation Influences the Performance of Dynamic Nuclear Polarization. *Angew. Chem. Int. Ed.* **2018**, 10.1002/anie.201800493.

4. Kress, T.; Che, K.; Epasto, L. M.; Kozak, F.; Negroni, M.; Olsen, G. L.; Selimovic, A.; Kurzbach, D., A novel sample handling system for dissolution dynamic nuclear polarization experiments. *Magnetic Resonance* **2021,** *2*, 387-394.

5. Baudin, M.; Vuichoud, B.; Bornet, A.; Milani, J.; Bodenhausen, G.; jannin, S., A Cryogen-Free 9.4 T System for Dynamic Nuclear Polarization. *under review.* **2018**.

6. Turhan, E.; Pötzl, C.; Keil, W.; Negroni, M.; Kouřil, K.; Meier, B.; Romero, J. A.; Kazimierczuk, K.; Goldberga, I.; Azaïs, T.; Kurzbach, D., Biphasic NMR of Hyperpolarized Suspensions─Real-Time Monitoring of Solute-to-Solid Conversion to Watch Materials Grow. *The Journal of Physical Chemistry C* **2023,** *127* (39), 19591-19598.

7. Schanda, P.; Forge, V.; Brutscher, B., Protein folding and unfolding studied at atomic resolution by fast two-dimensional NMR spectroscopy. *Proceedings of the National Academy of Sciences of the United States of America* **2007,** *104* (27), 11257-11262.

8. Schanda, P.; Kupce, E.; Brutscher, B., SOFAST-HMQC experiments for recording two-dimensional heteronuclear correlation spectra of proteins within a few seconds. *J Biomol NMR* **2005,** *33* (4), 199-211.

9. Schanda, P.; Brutscher, B., Very Fast Two-Dimensional NMR Spectroscopy for Real-Time Investigation of Dynamic Events in Proteins on the Time Scale of Seconds. *Journal of the American Chemical Society* **2005,** *127* (22), 8014-8015.

10. Ying, L.; Benjamin, W. J.; Chad, R. M., Selective refocusing pulses in magic-angle spinning NMR: Characterization and applications to multi-dimensional protein spectroscopy. *Journal of Magnetic Resonance* **2006,** *179* (2), 206-216.

11. Shaka, A. J.; Barker, P. B.; Freeman, R., Computer-optimized decoupling scheme for wideband applications and low-level operation. *Journal of Magnetic Resonance (1969)* **1985,** *64* (3), 547-552.

12. Szekely, O.; Olsen, G. L.; Felli, I. C.; Frydman, L., High-resolution 2D NMR of disordered proteins enhanced by hyperpolarized water. *Anal Chem* **2018**.

13. Kurzbach, D.; Canet, E.; Flamm, A. G.; Jhajharia, A.; Weber, E. M.; Konrat, R.; Bodenhausen, G., Investigation of Intrinsically Disordered Proteins through Exchange with Hyperpolarized Water. *Angew Chem Int Ed Engl* **2017,** *56* (1), 389-392.

14. Hilty, C.; Kurzbach, D.; Frydman, L., Hyperpolarized water as universal sensitivity booster in biomolecular NMR. *Nat Protoc* **2022,** *17* (7), 1621-1657.

15. Rule, G.; Hitchens, K., *Fundamentals of Protein NMR Spectroscopy*. 1st ed.; Springer, Dordrecht: 2006.
